# Oxygen‐Inhibition Driven Compartmentalization of Dextran Microgels: Toward Fusible Colloidal Biomaterial Inks for 3D Printing

**DOI:** 10.1002/adma.202519972

**Published:** 2026-03-14

**Authors:** Selin Bulut, Thomas Bissing, Tudor Lile, Hannah Küttner, Daniel Günther, Cédric Bergerbit, Dan Eugen Demco, Laura De Laporte, Andrij Pich

**Affiliations:** ^1^ DWI‐Leibniz Institute for Interactive Materials e. V. Aachen Germany; ^2^ Institute of Technical and Macromolecular Chemistry (ITMC) RWTH Aachen University Aachen Germany; ^3^ Department of Chemistry and Applied Life Sciences ETH Zurich Zurich Switzerland; ^4^ Advanced Materials for Biomedicine (AMB) Institute of Applied Medical Engineering (AME) Center for Biohybrid Medical Systems (CBMS) University Hospital RWTH Aachen Germany; ^5^ Aachen Maastricht Institute for Biobased Materials (AMIBM) Maastricht University Maastricht Netherlands

**Keywords:** 3D printing, bio‐based microgels, compartmentalized microgels, core–shell, dextran, microfluidics

## Abstract

This study presents a facile and versatile method to synthesize tailored, compartmentalized, polysaccharide‐based microgels, with ultra‐low crosslinked shells for the development of self‐setting colloidal biomaterial inks. Compartmentalization is achieved by exploiting spatially controlled oxygen‐inhibition of the crosslinking process in droplets obtained by droplet‐based microfluidics, leading to physically distinct core and shell regions inside dextran microgels. The shell exhibits a markedly lower cross‐linking density, resulting in reduced stiffness, tunable degradation, and higher macromolecular permeability than the core. The facile control of the core‐to‐shell ratio by varying the initiator concentration and oxygen availability represents a novel strategy to engineer microgel compartmentalization. This work establishes oxygen‐controlled photopolymerization as a new design principle for structuring microgels in flow, offering precise spatial control over polymer network architecture without complex templating or multi‐phase emulsions. Beyond dextran, this concept is broadly applicable to other acrylated and methacrylated hydrogel systems, opening new avenues for designing hierarchical soft materials. We demonstrate the applicability of these advanced microgels for the fabrication of millimeter‐sized tissue constructs, via 3D printing by exploiting the fusion of ultra‐low crosslinked shell compartments, thereby eliminating the need for additional chemical crosslinkers, initiators, or support baths to stabilize the final printed constructs.

## Graphical abstract

Harnessing oxygen inhibition as a materials design principle enables the universal fabrication of core–shell microgels with tunable radial crosslinking gradients. This conceptually simple yet highly versatile strategy generates fusible colloidal bioinks that combine structural hierarchy with adaptive porosity. The resulting microgel ink supports extrusion‐based 3D printing of dynamically remodeling scaffolds for advanced biofabrication and tissue engineering applications.

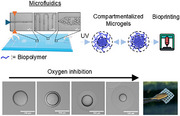

## 1 Introduction

Microgels are 3D crosslinked polymer colloids, which display unique attributes, determined by their porous nature and flexible structure, allowing them to absorb and retain significant amounts of a favorable solvent, such as water. This characteristic promotes a high mobility of solvents and solutes, such as metabolites or reactants, leading to physicochemical properties resembling biological materials. Through deliberate selection of starting materials and chemical functionalities, microgels can be designed to ensure biocompatibility and even possess biodegradable and stimuli‐responsive properties [1]. In addition, their size, morphology, and mechanical properties can be altered, making them attractive candidates for various biomedical applications, such as drug delivery or bioprinting [2, 3]. The growing concern over the environmental impact of synthetic polymers used to synthesize microgels, mostly derived from fossil resources, leads to an increased interest in bio‐based polymers, especially polysaccharides. Polysaccharides are naturally occurring polymers that consist primarily of monosaccharide units linked by glycosidic bonds [4]. Microorganisms can break down polysaccharides into harmless metabolites, making these materials suitable for biomedical applications. Most importantly, their chemical complexity enables facile modification for specific applications. An essential polysaccharide is dextran, which comprises an α‐(1,6)‐linked D‐glucose main chain with a small amount of mainly α‐(1,3)‐linked branches [5, 6]. Dextran formulations are frequently employed in pharmaceuticals and are considered an essential medicine by the World Health Organization [7]. Previously reported stable dextran‐based microgels and hydrogels have shown promise as candidates for tissue engineering applications [2]. To further expand the functionality of such systems, the ability to create different properties in a spatially controlled manner inside the microgels may become a key design parameter. In this context, the compartmentalization of microgels is highly important for many application fields that require complex architecture, such as defined pores, distinct patterns, or anisotropic structures. While porous polymer matrices are beneficial in drug loading and delivery, double or triple emulsions and resulting core–shell structured microgels are important for temporally controlled or sequential drug release and bio‐fabrication [8, 9]. Depending on the starting materials used, the core and the shell can exhibit different chemical functionalities, crosslinking densities, and stimuli‐responsiveness, which can lead to complex phase transition behaviors in response to diverse stimuli [10, 11].

Core–shell microgels can be synthesized through various established routes, depending on the desired morphology, composition, and colloid size. The most common strategy, precipitation polymerization, involves dissolving the monomer, initiator, and surfactant in water, followed by thermally triggered polymerization in the case of thermal initiators. After a certain reaction time, a second monomer is added, forming a crosslinked shell around the growing particle. This method yields small microgels with sizes ranging from a few to several hundred nanometers [12, 13]. A prominent example is the poly(*N*‐isopropylacrylamide)—*N*,*N*‐methylenebisacrylamide (pNiPAm‐BIS) microgel, where a core–shell structure is achieved through the occurrence of a radial gradient of the crosslinking degree due to the faster reaction kinetics of the crosslinking agent compared to NiPAm [14, 15]. Another method is seeded precipitation polymerization, where the precipitation polymerization is performed in the presence of rigid core particles to introduce the formation of a thick microgel shell [16].

However, traditional precipitation‐based methods for synthesizing core–shell microgels are typically confined to the sub‐micrometer size regime, which limits their use in cell‐laden or tissue‐scale applications. In contrast, droplet‐based microfluidics offers a facile, high‐throughput platform for producing larger monodisperse micron‐sized microgels with diameters ranging from tens to several hundreds of micrometers [17]. To produce core–shell particles via microfluidics, spatial separation within the droplet has been utilized. A common method used to create compartments is the fabrication of double emulsions within a microfluidic device. Double emulsions like O/W/O or W/O/W can be employed for capsule and core–shell microgel formation, but each system has drawbacks: in O/W/O emulsions, the precursor is located in the aqueous phase, and droplet generation requires a complex multistage microfluidic arrangement. Another major drawback is emulsion stability within the microfluidic device. To prevent channel wetting in a water/oil emulsion, the walls are hydrophobically functionalized, but in double emulsions, the surface wetting through the inner and middle phase has to be prevented, which necessitates complex, spatially specific surface modifications within the device [18].

To eliminate this challenge, a simpler solution was reported where polyethylene glycol diacrylate (PEG‐DA) and alginate were used as the pre‐polymers for the droplet formation. As PEG‐DA starts to crosslink, phase separation is induced, leading to a core‐shell morphology with a PEG core and an alginate shell [19]. Similarly, phase separation can be employed to produce water‐in‐water and even water‐in‐water‐in‐water droplets. Nevertheless, the interfacial tension between aqueous phases is very low, which makes these systems challenging to work with [20, 21].

To overcome the necessity of complex microfluidic devices, setups, synthetic polymers, and modifications to achieve compartmentalized microgels, we propose a synthesis method of biodegradable core–shell microgels made from a single biopolymer phase via droplet‐based microfluidics by exploiting oxygen inhibition of free‐radical polymerization of acrylated/methacrylated polymers and monomers [22]. While the presence of oxygen often leads to undesirable effects in the field of microfluidics and the production of thin films, it has also been used to control specific continuous polymeric fabrication processes. For example, the presence of oxygen‐rich regions have been used in stop‐flow lithography (SFL) and continuous liquid interface production (CLIP) polymer 3D printing to make use of the dissolved oxygen to compete with the photopolymerization reaction and create a zone where crosslinking is inhibited, avoiding clogging or sticking [23, 24, 25, 26]. In addition, this effect has been used to control the particle size by washing away unpolymerized shells [27].

**FIGURE 1:**
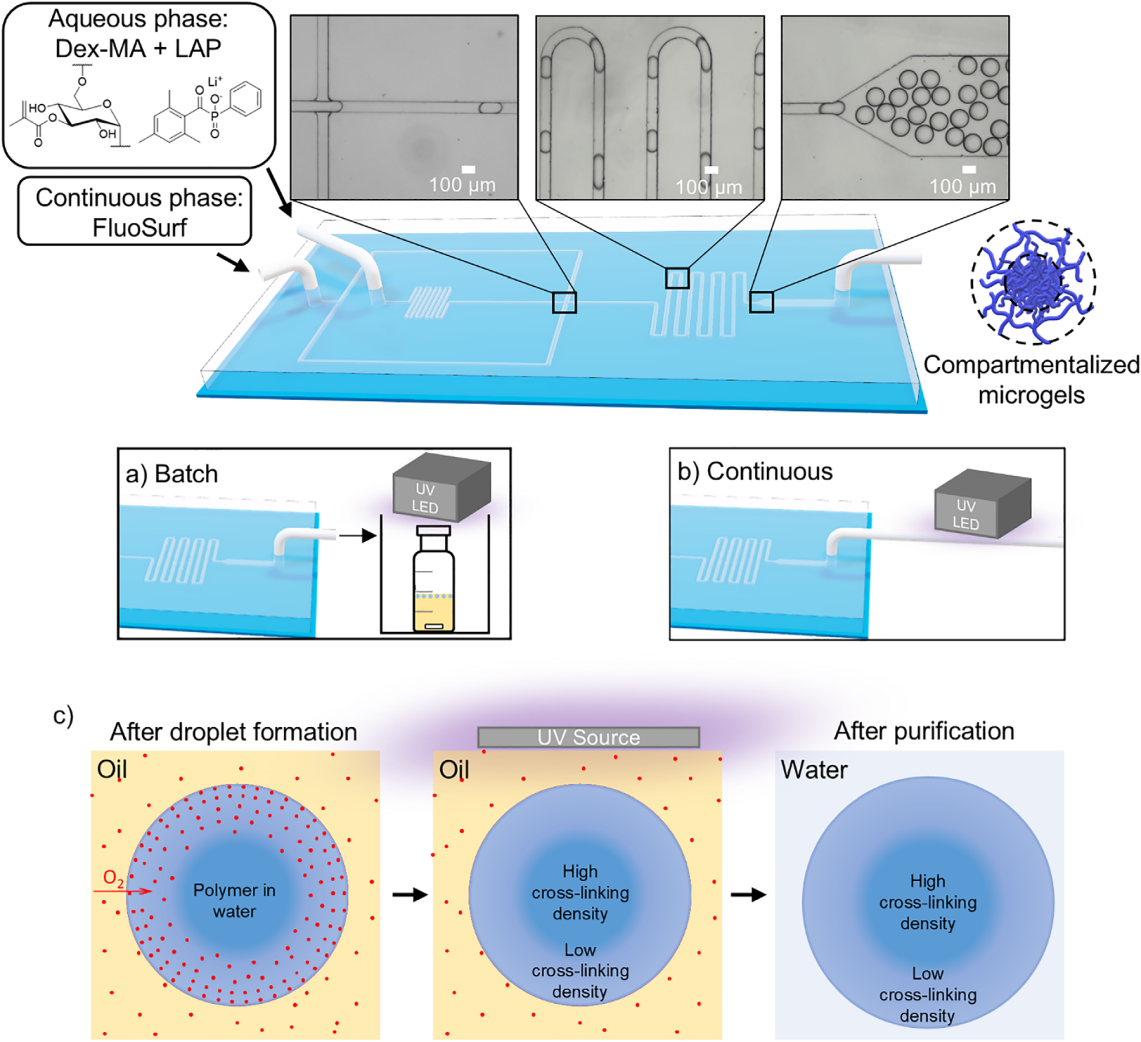
Scheme of a microfluidic device with microscopy images showing different channel regions during droplet formation, where the aqueous phase consists of dextran‐methacrylate (Dex‐MA) and Lithium phenyl‐2,4,6‐trimethylbenzoylphosphinate (LAP) in water, and the continuous phase: FluoSurf. (a) Off‐chip batch crosslinking scheme of the generated droplets and (b) Continuous on‐tube crosslinking procedure of the generated droplets. (c) Scheme of a droplet in fluorinated oil with dissolved oxygen (red dots) and a radial oxygen distribution (red gradient), undergoing crosslinking under UV irradiation, resulting in compartments (high crosslinked core and low crosslinked shell).

In our work, the effect of oxygen‐inhibited photo‐crosslinking is exploited to generate distinct compartments in a single microgel with different properties by inducing a more complete crosslinking of the core and an incomplete crosslinking of the shell. This yields an ultra‐low crosslinked shell compartment that is still stable after the purification process. The facile and controlled fabrication of core–shell microgels is achieved with a simple droplet‐generator microfluidic device. It requires no special equipment or techniques, such as double emulsions or phase separation, and can be achieved via an off‐chip UV exposure or a continuous on‐tube UV exposure procedure, while the oxygen diffusion rate can be controlled by the oxygen concentration inside the fluorinated carrier oil (Figure 1). The oxygen concentration in the droplets is determined by dissolved molecular oxygen in the fluorinated carrier oil. Under ambient conditions, the oil equilibrates with atmospheric oxygen. During photo crosslinking, oxygen is consumed within the droplets, generating a concentration gradient, enabling continuous diffusion of O_2_ into the droplets during photo crosslinking. The resulting microgels show promising results for 3D printing applications, with the lower crosslinked shell compartment being primarily used as the “glue” holding together the printed structures through interparticle fusion. This work demonstrates the facile synthesis of compartmentalized microgels, which is not limited to a specific polymer, and provides an extensive characterization of the compartments in terms of controllability, mechanical properties, permeability, and crosslinking density, showing its potential in the field of 3D printing.

## 2 Results and Discussion

### 2.1 Synthesis of Compartmentalized Microgels via Droplet‐Based Microfluidics

**FIGURE 2:**
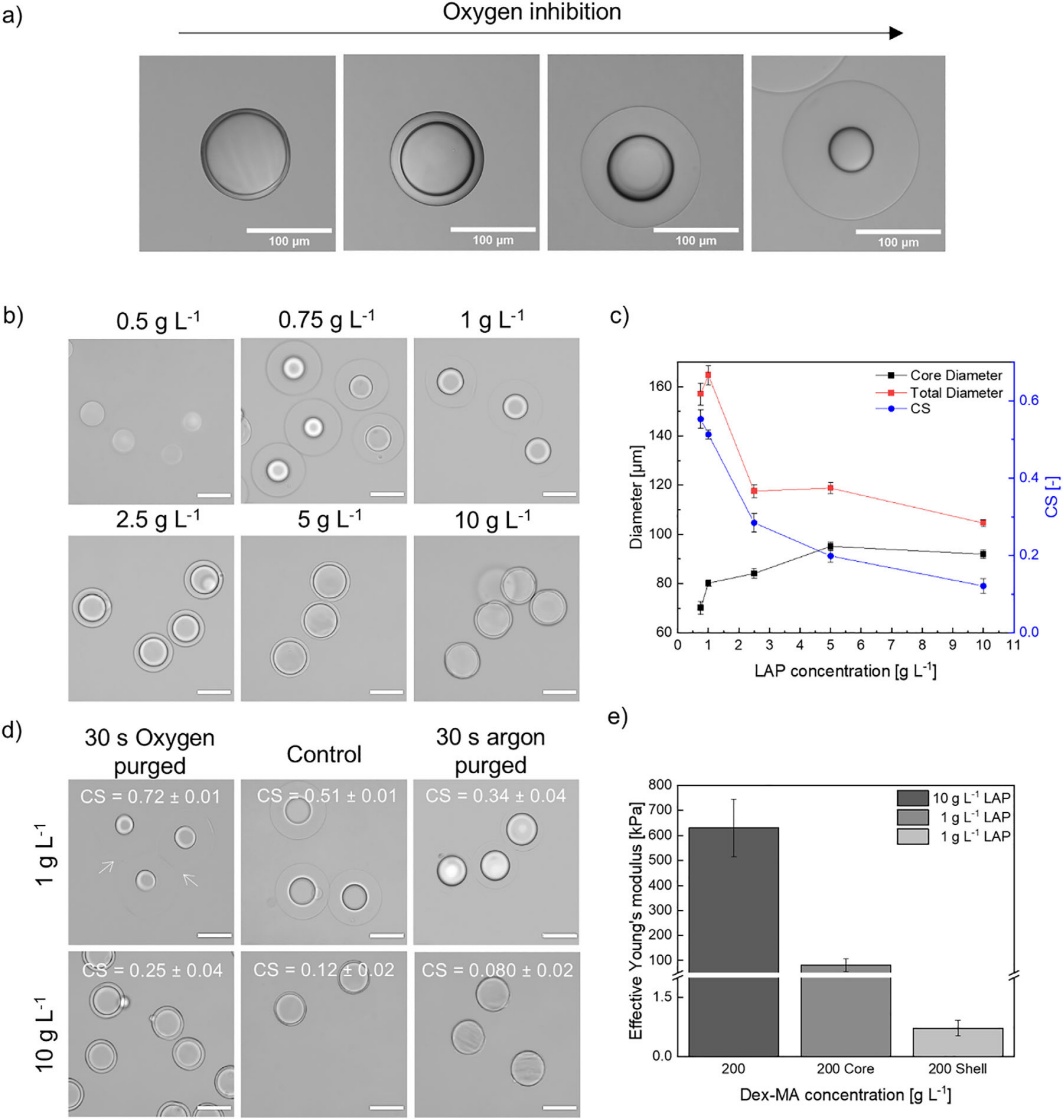
Microgels produced with the off‐batch crosslinking process. (a) Brightfield Microscopy images showing purified microgels with changing oxygen concentration during crosslinking. (b) Microscopy images of microgels in water after purification, synthesized with *c*(LAP) = 0.5 to 10 g L^−^^1^ and *c*(DexMA) = 200 g L^−^^1^, after 30 s of UV irradiation (365 nm). Scale bars represent 100 µm. (c) Graph of the core and total diameter of dex‐MA microgels in water with varying LAP concentration and resulting core–shell degree. (d) Microscopy images of microgels in water purged with oxygen during the cross‐linking process (left), non‐purged control (middle), and purged with argon during the cross‐linking process (right). White arrows point at the interface of the microgels in contact with each other. Scale bars represent 100 µm. (e) Young's moduli of microgels synthesized with 10 g L^−^^1^ LAP and core and shell of microgels synthesized with 1 g L^−^^1^ LAP.

Initially, droplet‐based microfluidics was used to form precursor‐filled aqueous droplets that were subsequently crosslinked by UV irradiation after collection in a container (referred to as the off‐chip batch crosslinking process in Figure 1a). The aqueous droplets contained dissolved reactive dextran chains along with the photoinitiator lithium phenyl‐2,4,6‐trimethylbenzoylphosphinate (LAP), which, upon UV irradiation, forms free radicals that initiate the polymerization process. The free radicals attack the methacrylate groups present on the dextran backbone, and the formed dextran macroradicals can recombine to form chains connected by covalent crosslinks. This free radical crosslinking process is very sensitive to the presence of oxygen molecules, which react with carbon‐centered radicals to form peroxy‐radicals, thereby converting highly reactive propagating species into less propagation‐efficient radicals. These peroxy‐radicals exhibit significantly reduced propagation rate constants toward acrylate and methacrylate double bonds, resulting in retardation of network formation rather than efficient formation of crosslinks [28, 29]. Oxygen molecules present in the fluorinated oil diffuse into the aqueous droplets, forming a concentration gradient with the highest oxygen concentration at the periphery of the droplets and the lowest oxygen concentration in the center [30]. Applying this scenario to our system, we confirmed our hypothesis that the enhanced inhibition of the free radicals leads to a smaller number of crosslinked dextran chains in the outer region of the droplet compared to the center. This effect was clearly visible through the formation of compartmentalized core–shell morphologies in the resulting microgels, featuring a highly crosslinked core and a loosely crosslinked shell depending on the oxygen concentration (Figure 2a).

We first investigated the influence of two parameters on the formation and tunability of the core–shell morphology for the off‐chip batch crosslinking process: the photoinitiator concentration and oxygen content.

#### 2.1.1 Influence of the Photoinitiator Concentration

The formation of two distinct compartments was found to be highly dependent on the photoinitiator concentration. When LAP concentrations in the aqueous phase were varied between 0.5 and 10 g L^−^^1^, microgels with different core–shell ratios were formed (Figure 2b). To quantify this ratio, a dimensionless number (CS) was introduced as a measure of the degree of core–shell formation (Equation 1).

(1) $$\mathrm{CS}=\frac{d_{T}-d_{C}}{d_{T}}$$

where *d*_C_ is the diameter of the microgel core, and *d*_T_ is the total diameter of the microgel.

A homogenous microgel without a shell (*d*_C_ = *d*_T_) would result in a CS value of 0, whereas a microgel with a small core (*d*_C_ ≪ *d*_T_) would result in a CS value approaching 1. Figure 2c shows a clear correlation between LAP concentration, microgel diameter, and CS. While a decrease in LAP concentration led to smaller core diameters and increased total diameters, an increase yielded larger core diameters but smaller total diameters. The highest LAP concentration of 10 g L^−^^1^ yielded CS of 0.12 ± 0.02, while the lowest LAP concentration of 0.75 g L^−^^1^ resulted in CS of 0.51 ± 0.01 with slight tendencies to form more than two visually distinct compartments due to low crosslinking density. Since all collected droplets produced by droplet‐based microfluidics are identical in size, this indicates an increased swelling of the microgels with lower LAP concentrations when introduced to water or phosphate‐buffered saline (PBS) post‐production. The increased swelling of the microgel shell at lower LAP concentrations is attributed to the reduced crosslinking density resulting from partial crosslinking due to oxygen inhibition. Stable partially crosslinked shells could not be obtained below 0.5 g L^−^^1^ LAP, limiting the maximum achievable CS in the off‐chip batch method. This observation is consistent with previous work, where the core–shell structure could be observed only in the emulsion by varying the LAP concentration from 1 to 5 g L^−1^ [27].

The reduced crosslinking density of the shell compartment not only results in increased swelling but also renders the shell selectively degradable. The degradation behavior of the core–shell microgels is investigated under different environmental conditions (Figure S1). Under physiological conditions (pH 7.4), the shell thickness gradually decreases over time, indicating progressive hydrolytic erosion of the less crosslinked outer compartment, while the highly crosslinked core remained structurally intact. Furthermore, exposure to alkaline environments (pH ≥ 8) accelerates shell degradation, while the highly crosslinked core compartment remains intact. Rapid shell degradation is demonstrated upon abrupt pH increase to 11 or enzymatic treatment with dextranase in Videos S1 and S2. In these cases, structural disintegration occurs within seconds to minutes. The degradation kinetics can therefore be tuned by adjusting the pH, enzyme activity, or crosslinking density of the shell, enabling stimulus‐responsive and compartment‐selective degradation.

To assess the mechanical properties of the microgels, nanoindentation experiments were performed for the microgels synthesized with the highest (10 g L^−^^1^) and lowest (1 g L^−^^1^) LAP concentrations by separately indenting the core and the shell compartments. To realize the separate measurement of the core compartment, the shell compartment was enzymatically degraded prior to the indentation. The reported effective Young's modulus for the microgels synthesized with the highest LAP concentration was 630 ± 120 kPa [31], while the effective Young's moduli for both the core and shell compartments of the microgels produced with a lower LAP concentration were much lower, with values of 82 ± 26 and 0.72 ± 0.20 kPa, respectively. These findings further support our assumption that the shell compartments are less crosslinked and therefore softer (Figure 2e).

#### 2.1.2 Influence of Atmospheric Conditions

The influence of oxygen concentration on the formation of core–shell microgels was investigated by adjusting the dissolved oxygen content in the fluorinated oil inside the crosslinking container using a syringe connected to an oxygen supply. A dex‐MA concentration of 200 g L^−^^1^ and LAP concentrations of 1 or 10 g L^−^^1^ were chosen for the synthesis of the core–shell microgels. The collected emulsions were purged with oxygen or argon for 30 s and subsequently crosslinked by exposure to UV light for 30 s, while the oxygen concentration inside the vial was continuously monitored with an oxygen meter (Table 1). After purging with oxygen, the dissolved oxygen level inside the fluorinated oil reached 43.15 ± 0.06 mg L^−^^1^, whereas purging with argon reduced it to 0.97 ± 0.05 mg L^−^^1^. A non‐purged control sample exhibited an intermediate oxygen concentration of 10.17 ± 0.02 mg L^−^^1^.

**TABLE 1:** Diameters of microgel samples with a high and low amount of LAP, purged with pure oxygen or pure argon during the cross‐linking procedure, and the control sample with calculated CS.

LAP concentration	Parameter	30 s oxygen purging	Control	30 s argon purging
1 g L^−^^1^	*c*O_2_ [mg L^−^^1^]	43.16 ± 0.09	10.16 ± 0.02	0.99 ± 0.03
	*d*_C_ [µm]	58.4 ± 4.9	80.2 ± 1.2	88.7 ± 3.6
	*d_T_* [µm]	187.5 ± 8.3	164.7 ± 3.8	135.0 ± 5.4
	CS	0.72 ± 0.01	0.51 ± 0.01	0.34 ± 0.04
10 g L^−^^1^	*c*O_2_ [mg L^−^^1^]	38.88 ± 0.10	9.71 ± 0.01	0.97 ± 0.22
	*d*_C_ [µm]	72.44 ± 4.09	92.0 ± 1.7	95.98 ± 2.55
	*d_T_* [µm]	100.07 ± 3.07	104.6 ± 1.4	104.41 ± 1.98
	CS	0.25 ± 0.04	0.12 ± 0.01	0.08 ± 0.02

As expected, the core sizes across all samples varied in diameter, demonstrating that the oxygen concentration had a significant impact on the crosslinking kinetics and the resulting core–shell morphology. This effect was most pronounced for the lower LAP concentration of 1 g L^−^^1^^,^ leading to microgels with the thickest shell and a CS value of 0.72 ± 0.01 at the highest oxygen concentration. Conversely, the lowest CS was observed in the case of argon purging at the higher LAP concentration (Figure 2c; Table 1). Notably, the cores of the oxygen‐purged microgels, prepared with 1 g L^−^^1^ LAP, were significantly smaller (58.4 ± 4.9 µm) than those of the control and argon‐purged samples. This can be attributed to the higher oxygen concentration in the oil phase, which promotes greater oxygen diffusion into the droplets. Since the depletion rate of oxygen is constant, oxygen can diffuse further into the droplet before it is fully consumed, resulting in a larger shell and a smaller core size of the microgel. It is noteworthy that these microgels with a large soft shell displayed a non‐spherical structure when in contact with other microgels (white arrows pointing at the interface between two microgels), likely due to the more loosely crosslinked shell and the resulting mechanical instability.

### 2.2 Morphological Characterization of Compartmentalized Microgels

#### 2.2.1 Permeability of Core and Shell Compartments

**FIGURE 3:**
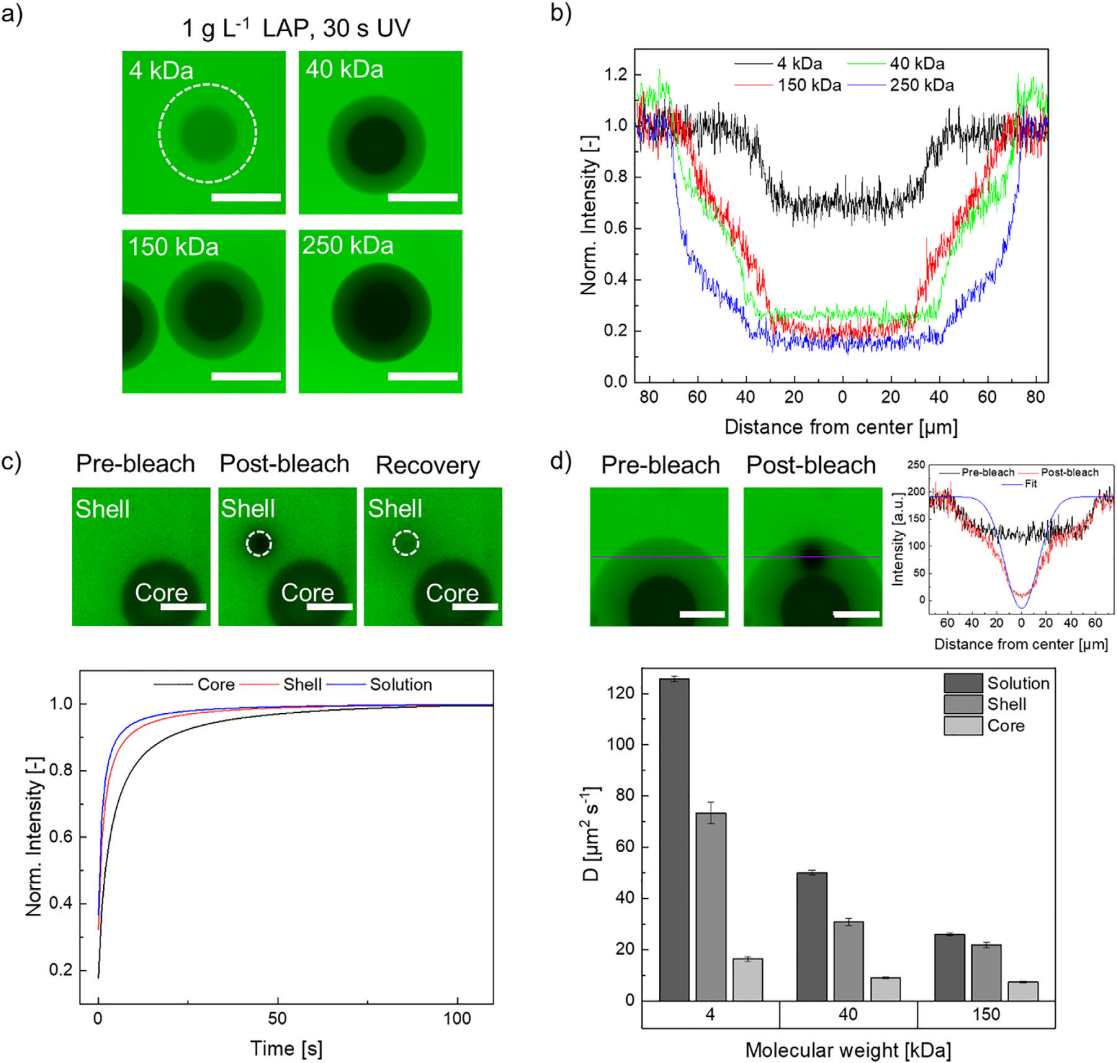
(a) Fluorescence confocal microscopy images of microgels synthesized with LAP concentrations of 1 g L^−^^1^immersed in FITC‐dextran solutions (*M*_w_ = 4, 40, 150, and 250 kDa) for permeability studies. Scale bars represent 100 µm. (b) Fluorescence intensity profiles of core–shell microgels in different FITC‐dextran solutions (4, 40, 150, and 250 kDa). (c) CLSM images of the FRAP process depicting pre‐bleach, bleach onset, and fluorescence recovery and fit functions of the fluorescence recovery of 4 kDa FITC‐dextran in the microgel core, shell, and in solution. (d) CLSM images of the bleach radius cross‐sections to determine the exact bleach radius from the fit function and calculated diffusion coefficients of 4, 40, and 150 kDa FITC‐dextran in different compartments of the core–shell microgels. Scale bars represent 50 µm.

To investigate the permeability of individual microgels compartments and visualize internal crosslinking gradients, control microgels (non‐gas‐purged) prepared with 1 g L^−^^1^ LAP were incubated for 24 h in aqueous solutions containing fluorescein isothiocyanate dextran (FITC‐dextran) of different molecular weights (4, 40, 150, 250 kDa). Since different molecular weights of FITC‐dextran correspond to different hydrodynamic radii, ranging from ∼1.3 to 15.9 nm, respectively, the permeability limits of the microgels provide important information on the mesh size and thus the crosslinking densities of each compartment [32]. After the incubation period, the core–shell microgels were analyzed by confocal laser scanning microscopy (CLSM) (Figure 3a).

The core–shell microgels with a CS of ∼0.51 showed higher fluorescence intensities within the shell than in the core, which verified our assumption that the shell is far less crosslinked than the core, translating to a higher porosity. Overall, the core compartment was limited to molecules with a hydrodynamic radius of ∼1.3 nm (4 kDa FITC‐dextran). In contrast, the shell compartment allowed diffusion of molecules with hydrodynamic radii up to tens of nanometers (see Figure 3a). Table 2 summarizes the relative fluorescence intensities of the core–shell microgels for different molecular weights of FITC‐dextran, calculated as the ratio of the mean fluorescence intensity within a compartment to that of the surrounding solution.

**TABLE 2:** Relative fluorescent intensities in the dex‐MA microgels' core and shell compartments immersed in different FITC‐dextran solutions.

FITC‐dextran (kDa)	*c*(LAP) (g L^−^^1^)	Rel. Intensity core (−)	Rel. Intensity shell (−)
4	1	0.693 ± 0.012	0.977 ± 0.007
40	1	0.226 ± 0.006	0.569 ± 0.007
150	1	0.223 ± 0.008	0.525 ± 0.021
250	1	0.144 ± 0.005	0.377 ± 0.012

Additional cross‐sectional fluorescence intensity profiles (Figure 3b) with 4, 40, 150, and 250 kDa revealed that the distribution of 4 kDa FITC‐dextran was homogeneously distributed within the shell compartment up to a certain gradient close to the core compartment, whereas higher molecular weight FITC dextrans (150 and 250 kDa) exhibited a wide gradual change in fluorescent intensity within the shell. This observation indicates the presence of a radial gradient in crosslinking density within the shell compartment, which is not directly discernible in microscopy images and does not markedly influence the motion of smaller molecules. These findings suggest that the shell consists of a mesh size gradient that cannot be fully visualized.

To obtain a better understanding of the diffusivity of molecules inside the core and shell of the microgels, fluorescence recovery after photobleaching (FRAP) was performed by bleaching a circular region of interest (ROI) of 20 µm with an argon laser. Fluorescence recovery was monitored over time, and the resulting curves were fitted according to a modified Soumpasis fit [33] (Figure 3c) for 4 kDa FITC‐dextran. While the fastest recovery occured in solution with a time constant (*τ*) of 0.849 ± 0.007 s, followed by the recovery of the microgel shell with *τ* = 1.25 ± 0.07 s, the slowest recovery occurred in the microgel core with a time constant of *τ =* 2.90 ± 0.17 s. These results show the similarity between the diffusion of FITC‐dextran inside the microgel shell compartment and in pure solution, elucidating the ultra‐low crosslinking density of the shell. The half‐width at half‐maximum (HWHM) radii of the bleach spot were used to determine the diffusion coefficient for each compartment (Figure 3d). The calculated diffusion coefficient of a 4 kDa FITC‐dextran solution in PBS is 125.77 ± 1.09 µm^2^ s^−^^1^, while the diffusion coefficient for the shell compartment is remarkably close to the diffusion coefficient in solution, with a value of 73.36 ± 4.15 µm^2^ s^−^^1^. In contrast, the core exhibited a markedly lower diffusion coefficient of 16.39 ± 0.94 µm^2^ s^−^^1^. A similar trend was observed for the higher molecular weight FITC‐dextrans of 40 and 150 kDa, which showed reduced diffusion coefficients of 50.08 ± 0.96 and 25.98 ± 0.59 µm^2^ s^−^^1^ in solution and 30.79 ± 1.42 and 21.85 ± 1.10 µm^2^ s^−^^1^in the shell compartment, respectively.

#### 2.2.2 Investigation of Core–Shell Morphology Using ^1^H T_2_ Relaxation

To investigate the influence of the UV exposure time on the crosslinking density of the compartmentalized microgels, microgels were prepared using the continuous on‐tube crosslinking procedure (1 g L^−^^1^, 5 and 77 s) and, for comparison, the batch procedure (10 g L^−^^1^, 1 h UV irradiation and 1 g L^−^^1^, 30 s). Different outlet tubing lengths (1 and 15 cm) were subjected to UV light, corresponding to exposure times of ∼5.1 and ∼76.6 s at a total flow rate of 800 µL h^−^^1^. Here, ^1^H T_2_ relaxation NMR was employed to study the core–shell morphology. The proton transverse magnetization relaxation (T_2_ relaxation) of the hydrogen nuclei offers valuable insights into the morphology of various materials and helps gain further understanding of differences in crosslinking density. In polymers, the degrees of freedom of the protons are restricted, resulting in the T_2_ transverse relaxation of ^1^H being mostly governed by interactions with the local environment. Consequently, higher crosslinking densities in hydrogels yield shorter T_2_ relaxation times due to stronger dipolar‐spin interactions [34]. Structural inhomogeneities of core–shell microgels obtained from precipitation polymerization were intensively studied via ^1^H NMR transverse relaxation in the past, where the material morphology determined by the crosslinking density was analyzed via T_2_ relaxation time measurements.

**FIGURE 4:**
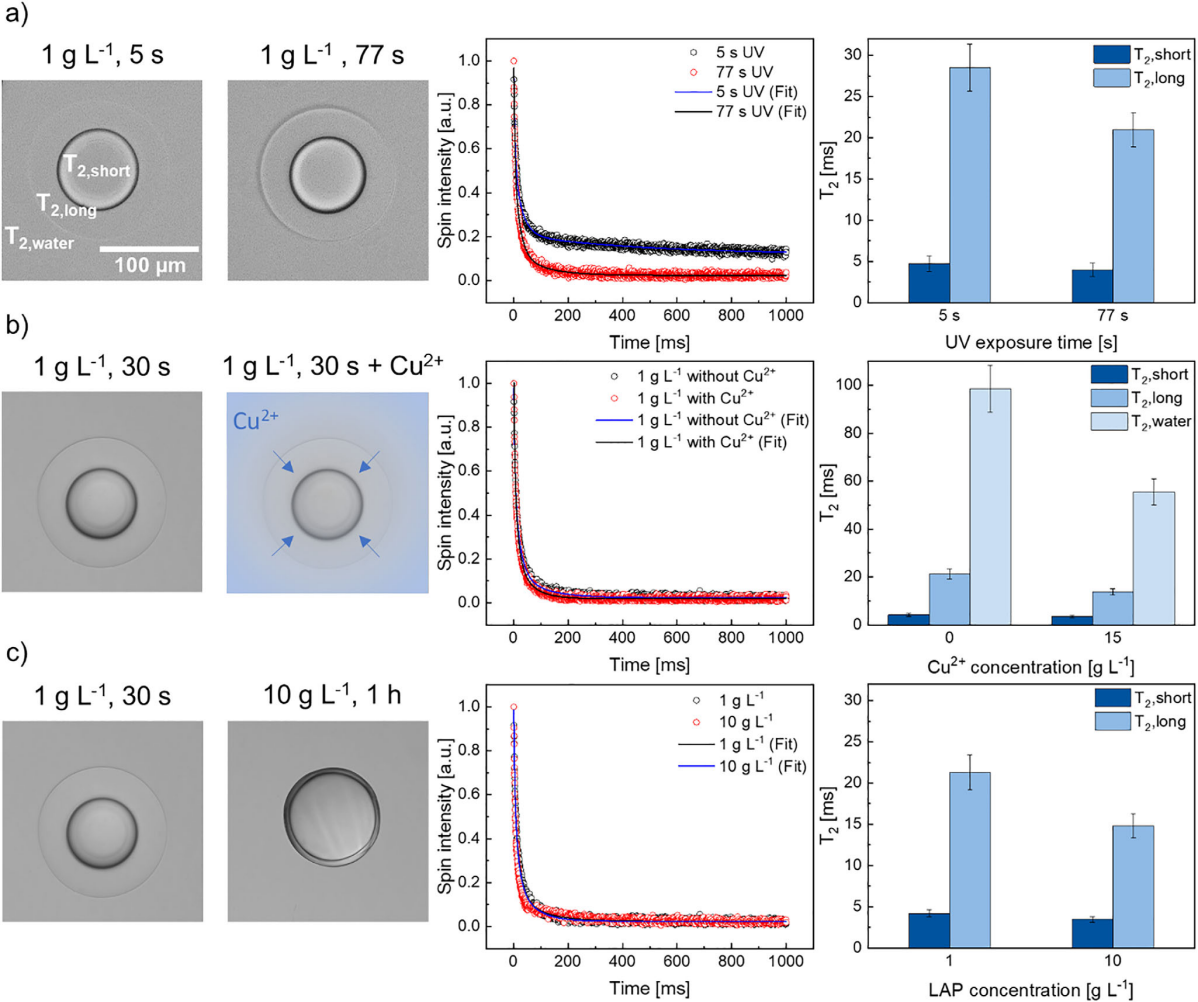
Brightfield microscopy images, CPMG decay curves, and T_2_ relaxation times of microgels synthesized with (a) 1 g L^−^^1^ LAP and irradiated for 5 and 77 s, (b) 1 and 1 g L^−^^1^ + Cu^2+^ irradiated for 30 s, and (c) 1 and 10 g L^−^^1^ irradiated for 30 s and 1 h, respectively.

The microgel samples were fabricated with 1 g L^−^^1^ LAP according to the standard protocol, but water was replaced by D_2_O for the aqueous phase. As both UV exposure times resulted in microgels without a visible difference, the T_2_ relaxation times were determined for the different compartments. Both UV exposure times produced visually identical microgels, yet the spin‐echo decays revealed clear differences in ^1^H transverse magnetization (Figure 4a). A notably faster Carr‐Purcell‐Meiboom‐Gill (CPMG) decay was observed for the sample irradiated for ∼77 s compared to the sample irradiated for a shorter time (∼5 s), which is caused by increased residual dipole interactions for higher crosslinked polymers. Through fitting the CPMG decay, the T_2_ relaxation times could be calculated. Two significantly different T_2_ relaxation times were observed, associated with the hydrogen nuclei within the polymer matrix (Figure 4a). The shorter T_2_ time (T_2_,_short_) corresponds to the more densely crosslinked region (core), while the longer T_2_ time (T_2_,_long_) is associated with a less crosslinked area (shell). While there is no pronounced reduction of the T_2_,_short_ times (core) for irradiation times ranging from 5 to 77 s, measured to be 4.70 ± 0.94 and 3.97 ± 0.79 ms, respectively, the T_2_,_long_ times (shell) showed a large disparity with 28.5 ± 2.9 and 21.0 ± 2.1 ms, respectively. This indicates that the crosslinking density of both compartments can be varied independently, as the shell properties were more affected by the UV exposure time than the core. We attribute this difference to a higher oxygen concentration near the shell, which locally inhibits free‐radical polymerization and results in a loose network. To further assess the early‐stage kinetics of compartment formation, additional microgels were produced in batch under short UV irradiation times ranging from 1 to 30 s (Figure S2). The resulting core–shell morphologies exhibited nearly constant CS values of ∼0.44–0.47 up to 5 s, followed by a slight increase to ∼0.5 at 30 s. The T_2_,_long_ for 30 s UV exposure in the batch process, is similar to the T_2_,_long_ for ∼77 s UV irradiation in the continuous process. This plateau behavior indicates that the core–shell structure is established within the first seconds of irradiation and that its dimensions remain largely unaffected by further exposure, consistent with a rapid oxygen‐limited regime that self‐stabilizes once the diffusion‐reaction balance is reached. The increase observed at 30 s suggests minor additional densification of the shell, in agreement with the trends obtained from T_2_ relaxation analysis.

To verify whether the T_2_,_long_ time accounts for the shell compartment, a paramagnetic relaxation enhancement experiment was employed to alter the relaxation rate of the protons by introducing Cu^2+^ ions into the system (Figure 4b and Table 3). Microgels were again produced using the batch process with 1 g L^−^^1^ LAP and a UV exposure time of 30 s. Since the concentration of Cu^2+^ ions was highest in the D_2_O solvent and lower in the shell and core compartments due to diffusion, the effect of adding Cu^2+^ on the T_2_ relaxation times was strongest in the solvent, with a relative relaxation decrease of 44%. The differences in T_2_ with and without the addition of Cu^2+^ allowed us to assign the T_2_ relaxation times to the respective compartments, with relative relaxation decreases of 35% and 19% in the shell and core compartments, respectively. This verifies that the longer T_2_ times correspond to the shell compartment, whereas the shorter T_2_ times correspond to the core compartment.

**TABLE 3:** T_2_ relaxation times of microgels immersed in D_2_O and D_2_O with Cu^2+^ ions and their relative change ΔT_2_.

Parameter	0 g L^−^^1^ Cu^2+^	15 g L^−^^1^ Cu^2+^	ΔT_2_ [%]
T_2, short_ [ms]	4.15 ± 0.62	3.36 ± 0.50	19
T_2, long_ [ms]	21.3 ± 2.1	13.8 ± 1.4	35
T_2, D2O_ [ms]	98.6 ± 9.9	55.5 ± 5.6	44

To evaluate whether the initiator concentration affects the relaxation times, the same microgels were produced with two different LAP concentrations, 1 and 10 g L^−^^1^ (Figure 4c). Because the 10 g L^−^^1^ LAP sample was irradiated longer (1 h, batch process) than the 1 g L^−^^1^ samples (30 s, batch process), LAP concentration and UV exposure time were not fully decoupled. The high‐initiator, long‐irradiation condition was intentionally included to probe the upper limit of crosslinking and define the boundaries of the system. Nevertheless, the decrease of T_2_,_long_ observed at 10 g L^−^^1^, 1 h is most likely more strongly affected by the higher initiator concentration, which increases radical density and accelerates shell crosslinking, rather than by the UV exposure time. Images of microgels produced with 10 g L^−^^1^ LAP for 30 s or 1 h did not reveal significant changes in morphology, and both microgels had an average CS value of 0.12 ± 0.01 (Figure S3). This indicates that UV exposure dominates at short times while initiator availability becomes increasingly influential at longer irradiation times. Importantly, no pronounced changes were observed in T_2_,_short_ when altering the initiator concentration or UV exposure time, confirming that variations in polymerization conditions primarily affect the oxygen‐rich shell region.

## 3 Application of Compartmentalized Microgels as Biomaterial Inks for 3D Printing

The unique controllable morphology of the dextran‐based microgels produced in this report makes them excellent candidates for use in 3D printing as granular ink. The shell compartments can interlink after extrusion through physical chain entanglement, stabilizing the printed structure without the need for external crosslinkers or UV irradiation, usually applied to stabilize the final printed structure. In addition, this method enables the fabrication of various stable 3D structures without the need for additional chemical reactive groups.

**FIGURE 5:**
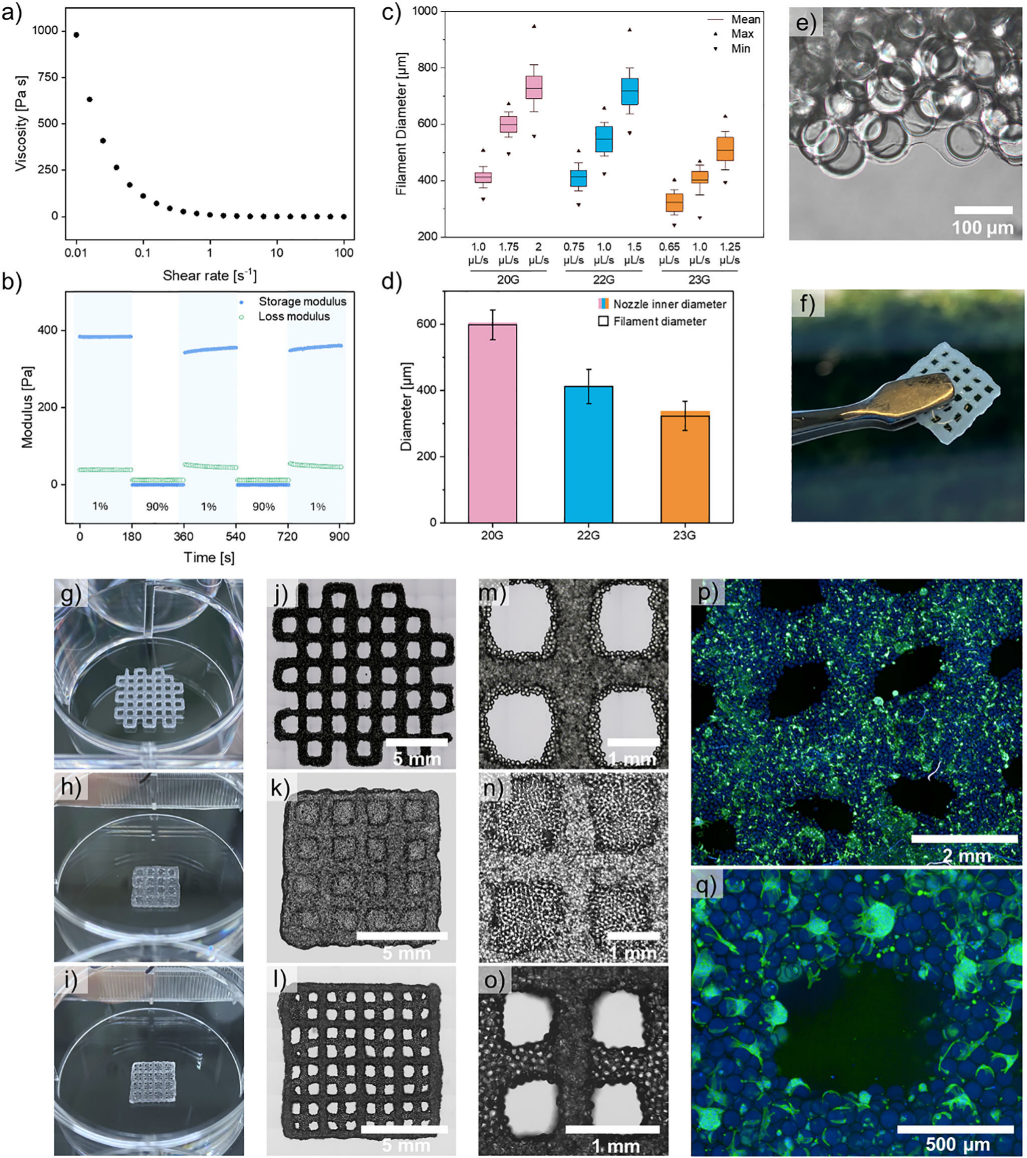
(a,b) Rheological measurements of the microgel ink. (a) Shear rate sweep measurement with shear rates from 0 to 100 s^−^^1^ at 1% strain. (b) Step‐strain sweep with alternating strains of 1% and 90% cycled every 180 s at a frequency of 1 Hz. (c) Quantitative analysis of filament diameters obtained using different nozzle sizes (20, 22, 23G) at different extrusion flow rates. (d) Comparison of the nozzle inner diameters to the resulting filament diameter at optimal extrusion flow rates. (e) Brightfield image of the annealed colloidal biomaterial ink reswollen in PBS. (f) printed 4‐layer construct held by tweezers. (g—o) Images of the printed constructs in a 6‐well plate with different geometries and flow rates. (g) Open‐edged two‐layer geometry printed with a 22G nozzle with a flow rate of 1.5 µL s^−^^1^. (h) Close‐edged two‐layer geometry printed with a 22G nozzle and a flow rate of 1 µL s^−^^1^. (i) Close‐edged two‐layer geometry printed with a 22G nozzle and a flow rate of 0.75 µL s^−^^1^. (j—l) Brightfield microscopy images of the printed constructs with different geometries and high magnification images. (m—o) Magnified images of printed constructs (p) Confocal laser scanning microscopy (CLSM) images (z‐stack projection) of a construct with open‐edged geometry printed with a flow rate of 1.5 µL s^−^^1^, incubated with 4 × 10^6^ NHDFs for 2 days. (q) CLSM close‐up image of a construct with closed‐edged geometry printed with a flow rate of 0.75 µL s^−^^1^, incubated with 2 × 10^6^ NHDFs after 4 days.

Rheological characterizations of the jammed microgels were conducted to evaluate the printability of the core–shell microgels. Shear rate sweep experiments verified shear‐thinning behavior (Figure 5a). Strain sweep measurements showed that the core–shell microgels can quickly recover from applied strain upon strain removal (Figure 5b).

The printability and the printing fidelity of the microgel ink were tested with a commercial extrusion‐based 3D bioprinter using varying extrusion speeds and nozzle dimensions. The operating window is defined as the parameter space in which continuous, self‐supporting, and well‐separated filaments are obtained. Straight filaments were printed at a constant speed of 6 mm s^−^^1^ to determine the optimal extrusion flow rates and printing fidelity (Figure 5c). Filament fidelity decreased at higher extrusion flow rates due to filament build‐up, and filament breakage was observed at flow rates that were too low. At optimal flow rates (1.75, 0.75, or 0.65 µL s^−^^1^ for 20, 22, and 23G, respectively), the resulting filaments exhibited uniform thickness and high printing fidelity with filament diameters almost identical to the nozzle's inner diameter (Figure 5d). The shell compartments of the printed core–shell microgels annealed after drying and subsequent reswelling in water, PBS, or culture medium, thereby stabilizing the printed geometry (Figure 5e; Video S3). For tissue engineering applications, the constructs must remain stable in aqueous environments at least until cellularization takes place. The stability against degradation and external mechanical stress was demonstrated by incubating the constructs in culture medium for several days and vigorously shaking the printed constructs in water (Video S4). Additionally, the constructs can be handled with a pair of tweezers without disrupting their structural integrity (Figure 5f).

In Figure 5g–o, we illustrate the precise control over the printed architecture that can be achieved by printing more complex constructs that have a high shape fidelity with their CAD models (Figure S4). Before evaluating cellular behavior within printed structures, the cytocompatibility of the microgels themselves was assessed by Live/Dead staining of normal human dermal fibroblasts (NHDFs) cultured with individual microgels (Figure S5). The NHDFs exhibited high viability of 97.6 ± 1.1% after 1 day and 98.8 ± 0.4% after 3 days, confirming that the core–shell microgels do not induce cytotoxic effects. Additionally, we demonstrate the biocompatibility and the potential application of the printed material in tissue engineering by seeding NHDFs onto the printed structures (Figure 5p,q). Constructs composed of two stacked layers of microgel‐based biomaterial ink, printed with a flow rate of 0.75 and 1.5 µL s^−^^1^, were chosen for the cell experiments. The confocal images validate the successful cellularization of the constructs within a few days. Cell adhesion and spreading were observed on and within the printed constructs, predominantly along microgel boundaries, leading to millimeter‐sized tissue constructs, while Live/Dead staining of the cells within the printed constructs confirms their viability (Figure S5).

**FIGURE 6:**
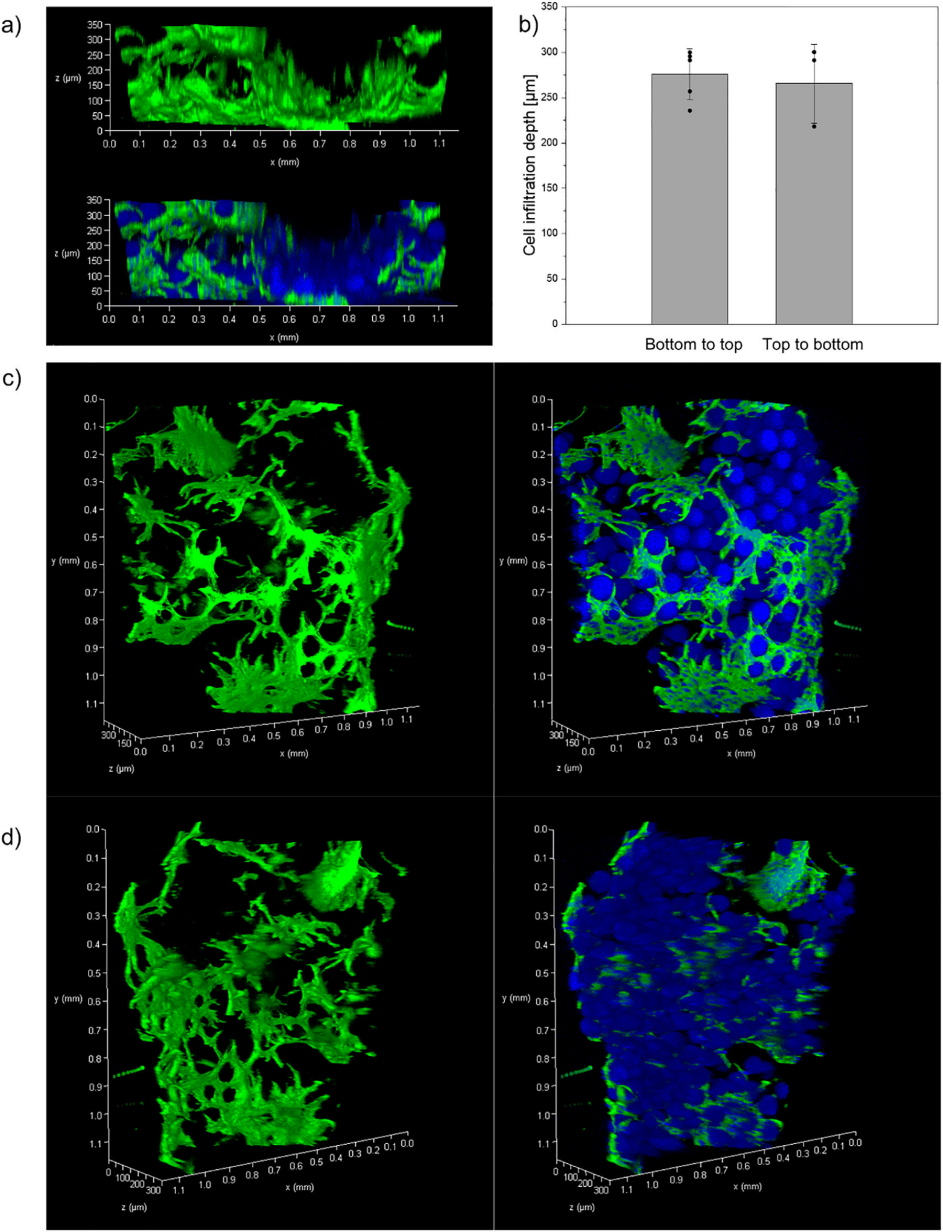
NHDF infiltration into 3D printed core–shell microgel scaffolds after 4 days of incubation time. (a) Orthogonal XZ projections of phalloidin‐stained NHDFs (green), cell nuclei and microgels (blue), and merged channels, revealing cell penetration in the interior of the construct. (b) Quantification of maximum cell infiltration depth determined from orthogonal projections (*n* = 5 independent measurement positions) for both orientations. Representative 3D volume rendering. (c) (top view) and (d) (bottom view) illustrating cellular distribution within the interconnected pore architecture of the printed scaffold. *z* = 0 represents the top of the scaffold, and *z* > 0 represents the middle of the scaffold. The total scaffold depth is 600 µm.

To further assess cellular infiltration into the printed core–shell microgel scaffolds, confocal z‐stack imaging was performed after 4 days of culture. Orthogonal XZ projections reveal homogeneous infiltration of NHDFs into the construct interior (Figure 6a). Cells were seeded onto the top surface of the printed constructs. Confocal imaging was performed using an inverted Leica TCS SP8 system. Due to the intrinsic optical penetration limit of confocal microscopy (∼300 µm under the chosen conditions), scaffolds were imaged from both orientations. The samples were inverted to enable imaging from the originally seeded surface (top part of the construct). In both orientations, pronounced cellularization is observed. Here, the infiltration depth is defined as the distance from the scaffold top (*z* = 0) to the last detectable phalloidin signal exceeding background intensity (8.4 ± 4.3). Based on this threshold‐based analysis, the mean infiltration depth is 265.45 ± 43.42 µm, indicating substantial cellular infiltration within the scaffold.

When imaging from the bottom surface, a pronounced phalloidin signal is detected, likely due to the cells that settled on the bottom during cell seeding (Figure S6, Video S5). Here, cells were observed up to 275 ± 28.2 µm from the bottom, when considering the last detectable phalloidin signal exceeding background intensity (77.5 ± 8.26).

The observed distribution, therefore reflects a combination of vertical infiltration through the interconnected porous architecture and lateral migration of the cells that settled to the bottom during cell seeding.

Throughout the scaffold, the cells exhibit pronounced elongated morphologies (Figure 6c,d), and Video S6 further indicates active adhesion and spreading rather than passive sedimentation. These observations suggest that the printed core–shell microgel architecture provides sufficient mechanical stability for cell attachment while maintaining an interconnected porous network that enables cellular migration.

The gradual erosion of the shell compartment under physiological conditions may further contribute to the observed cellular infiltration. As the less crosslinked shell progressively decreases in thickness over time, the void spaces within the printed construct may increase, facilitating enhanced cell migration and interconnection between adjacent microgels, while preserving the mechanically stable core framework and overall construct integrity. A gradual degradation of the shell component can be observed when comparing images of printed structures before and after cellular culture for 4 days (Figure S7).

## 4 Conclusion

In this study, we introduce a novel method for the compartmentalization of biocompatible and biodegradable polysaccharide microgels based on an oxygen‐driven inhibition of the crosslinking process in droplets obtained via microfluidics. Furthermore, we demonstrate that these compartmentalized microgels can be effectively utilized as colloidal biomaterial ink for tissue engineering applications. The microgels obtained with this method were extensively characterized by microscopy techniques, permeability assays, nanoindentation measurements, and T_2_‐relaxation NMR. By varying the photoinitiator concentration and atmospheric conditions, different compartmentalization degrees could be achieved. Permeability experiments revealed a diffusion gradient of FITC Dextran within the microgel, indicating a radial crosslinking gradient. This implies the presence of subcompartments and interfaces that are not detectable in microscopy images. Overall, the shell compartment was shown to be highly permeable toward larger FITC‐dextran molecules due to its ultra‐low crosslinking degree. The two visually distinct compartments are shown to be independently tunable from one another by varying UV exposure times, confirmed by T_2_‐relaxation NMR. Rheological characterization of the jammed colloidal suspension revealed its shear‐thinning behavior, and its printability was demonstrated to be dependent on the extrusion flow rate. After 3D printing of the microgels as colloidal biomaterial inks, we showed that the low crosslinking properties of the shell compartments allowed for the interlinking of microgels upon drying and reswelling without the need for secondary crosslinking to form stable 3D constructs. Post‐printing cellularization confirmed that the printed constructs support cell attachment and infiltration, enabling the fabrication of millimeter‐sized tissue constructs. Notably, the controllable gradual degradation of the less crosslinked shell compartment likely introduces a dynamic increase in accessible void space within the scaffold. This dynamic structural adaptation distinguishes the present system from rigid microgel networks with static architectures, as it may enable progressive cellular migration and interconnection while maintaining mechanical stability throughout the network. In summary, compartmentalized microgels with ultra‐soft shells exhibit favorable characteristics as colloidal biomaterial inks for 3D printing and offer a broadly applicable route toward hierarchical bio‐fabrication materials.

## 5 Experimental Section

### 5.1 Materials

Antibiotic‐antimycotic (ABM), Gibco Life Technologies, Aquapel (PPG industries), Bovine serum albumin (BSA, Sigma Aldrich), Copper sulfate (Sigma Aldrich), 4’,6‐Diamidino‐2‐phenylindole (DAPI, Thermo Fischer Scientific), Deuterium oxide (D_2_O, 99.90% Deutero), Dextran (Dextran100, Carl Roth), Glycidyl methacrylate (GMA, ≥97.0% GC, Sigma Aldrich), 4‐Dimethylaminopyridine (DMAP, ≥99%, Sigma Aldrich), Dimethyl sulfoxide (DMSO, 100%, VWR Chemicals), Dulbecco's modified eagle serum (DMEM, Gibco Life Technologies), Fetal calf serum (FCS, Capricorn Scientific), Fluorescein isothiocyanate‐dextran (Mn = 4 kDa, Sigma Aldrich), Fluorescein isothiocyanate‐dextran (Mn = 40 kDa, Sigma Aldrich), Fluorescein isothiocyanate‐dextran (Mn = 150 kDa, Sigma Aldrich), Fluorescein isothiocyanate‐dextran (Mn = 250 kDa, Sigma Aldrich), Fluorescein isothiocyanate‐dextran (Mn = 500 kDa, Sigma Aldrich), FluoSurf (Emulseo), GRGDSPC (RGD, CPC Scientific Inc.), Heptane (>99%, Sigma Aldrich), Hydrofluoroether Novec 7500 (3 M), LIVE/DEAD cell imaging kit (Invitrogen), Lithium phenyl‐2,4,6‐trimethylbenzoylphosphinate (LAP, > 95%, Sigma Aldrich), Paraformaldehyde (PFA, AppliChem), Phalloidin‐iFLuor 488 (Abcam), Phosphate buffered saline (Gibco Life Technologies), Primary normal human dermal fibroblast (NHDF, Promocell), Sylgard 184 Silicone elastomer kit (Dow Corning Inc.), Triton X‐100 (NanoEnTek Inc.), Water (HPLC grade, Sigma Aldrich).

### 5.2 Microfluidic Device Fabrication

A master mold prepared in previous works by soft lithography was used to produce microfluidic chip devices [35]. PDMS and a crosslinker were mixed in a ratio of 10 parts PDMS to 1‐part crosslinker. This mixture was placed in a desiccator and degassed three times for 15 min each time to remove any air bubbles. After degassing, the mixture was poured slowly into a master mold, and any remaining air bubbles were removed by degassing the PDMS‐filled mold again. Using a syringe cannula, any leftover bubbles were eliminated. The filled master mold was then cured overnight at 60°C to allow the PDMS to harden completely. Once cured, the PDMS was carefully removed from the master mold. Next, a biopsy punch with a diameter of 0.75 mm was used to create holes for inlet and outlet tubing. The PDMS chip and a glass slide were sonicated in 2‐propanol for 20 min and then washed with 2‐propanol and distilled water. They were dried using compressed air between each washing step. This cleaning process was repeated three times to ensure thorough cleanliness.

### 5.3 Functionalization of Dextran

Dextran functionalization was adapted from van Dijk‐Wolthuis et al. [36]. The synthesis was adapted from previous work and performed under oxygen‐ and water‐free conditions in a round‐bottom flask [31]. Dextran (20 g) with a molecular weight of 100 kDa was dissolved in anhydrous dimethyl sulfoxide (DMSO, 190 mL). The solution was flushed with nitrogen for 30 min. After complete dissolution of dextran, dimethyl aminopyridine (DMAP, 4 g) was added. The reaction was started by the addition of glycidyl methacrylate (GMA, 13.05 mL). The solution was stirred at room temperature for 48 h. After the reaction was complete, 3.5 mL of HCl was added, and the reaction mixture was dialyzed against distilled water at 4°C. After one week of dialysis, the solution was lyophilized for three days, and the dextran methacrylate was stored at −20°C. The achieved functionalization degree was 37%, determined by ^1^H NMR spectroscopy via integration of the respective signals (Figure S8).

### 5.4 Synthesis of Dextran‐Based Core–Shell Microgels

The following microfluidic syntheses were adapted from previous work [31]. Dextran methacrylate (200 mg, 200 g L^−^^1^) and LAP (1 mg, 1 g L^−^^1^) were filled into a vial and filled with 1 mL of water (HPLC grade). The vial was wrapped in aluminum foil to protect the photoinitiator from ambient light. The solution was put on a shaker for 30 min to ensure that all reagents were dissolved. The fully dissolved solution was transferred to a 1 mL Hamilton glass syringe. Analogously, the syringe was wrapped in aluminum foil. The syringe cannula was inserted into the inlet tubing (40 cm, 0.38 mm inner diameter), surrounded by a larger, black tubing to hinder early crosslinking. A second 5 mL Hamilton syringe was filled with FluoSurf, and both syringes were mounted onto syringe pumps. Microfluidic experiments are conducted on a microfluidic station consisting of two syringe pumps (PHD Ultra, Harvard Apparatus, Holliston, U.S.A.) and an inverted microscope (Olympus CKX53) equipped with a camera (Olympus DP23), which is used to observe the flow and droplet formation. The flow rate for the aqueous phase was set to 200 µL h^−1,^ and the flow rate for the continuous oil phase was set to a flow rate of 600 µL h^−1^. After continuous uniform droplet formation, the outlet tubing was connected to the microfluidic device to collect the droplets, or the outlet tubing was irradiated for continuous production, and the resulting microgels were collected. Microchannels with a rectangular cross‐section of a uniform height of 80 µm are used for the microfluidic syntheses.

### 5.5 3D Printing

3D extrusion printing was performed with a commercial printer (REGENHU 3D Discovery). Prior to printing and cellularization experiments, the microgels were incubated with the adhesive peptide GRGDSPC to promote integri‐nmediated cell adhesion. The microgels were jammed by evaporating residual water on a heated glass plate at 50°C and subsequently filled in a cartridge consisting of a modified syringe (Hamilton 1000 Gastight) with sterile high‐precision blunt needles ranging from 23G (inner diameter 0.337 mm) to 20G (inner diameter 0.603 mm). All constructs were directly printed onto VWR 6‐well cell culture plates at a printing speed of 6 mm s^−^^1^. The extrusion flowrates and printhead parameters were controlled with the REGENHU HMI software. The geometry of the constructs was designed using the BioCAD software.

### 5.6 Characterization Methods

#### 5.6.1 Brightfield Microscopy

Microscopy images of the microgels were recorded using an inverted microscope (Olympus CKX53) equipped with a camera (Olympus DP23). The software Olympus cellSens Standard 3.2. Software was used to preview and save images. Additionally, tile scans of the constructs were acquired on an Echo Revolution microscope. Objectives ranging from 4x to 40x magnification were used.

#### 5.6.2 Confocal Laser Scanning Microscopy

Fluorescence and brightfield images of the microgels were recorded using a confocal microscope, Leica TCS SP8 (Leica, Wetzlar, Germany) with an Objective HC PL FLUOTAR 10x/0.30 (dry). The fluorophore was excited with an Argon laser 488 nm (20%, 10% intensity), and the fluorescence intensity was detected with a photo‐multiplier tube (PMT) detector (range: 500–550 nm). For brightfield images, a PMT trans detector was used.

The cellularized constructs in Figure 5 were imaged using a PerkinElmer Phenix Plus with a 5×/N.A. 0.16 air objective or a 10×/N.A. 0.3 air objective. The cellularized constructs in Figure 6 were recorded using the confocal microscope Leica TCS SP8 for orthogonal projections and 3D renderings of cellularized constructs. Quantification of maximum cell infiltration depth was determined from orthogonal projections (*n* = 5 independent measurement positions). Infiltration depth is defined as the maximum distance from the scaffold bottom or top (*z* = 0) to the last detectable phalloidin signal exceeding background intensity.

#### 5.6.3 Fluorescence Recovery after Photobleaching

FRAP experiments were performed using the CLSM setup. The microgel samples were placed on a microscopy slide with a press‐to‐seal silicone isolator and closed with a cover slip to prevent evaporation during measurement. Per sample, 28 measurements were conducted, 12 each for the core and the shell of the microgel, and 4 measurements for the solution. FITC excitation was achieved with an Argon laser line of 488 nm. The fluorescence intensity was measured in the range of 500 to 550 nm using a PMT detector. The samples were bleached with a laser intensity of 80%, and the bleached region of interest was set to 20 µm in diameter. To correct for changes in fluorescence due to unwanted bleaching effects or evaporation, the mean fluorescence of three different ROIs was measured over time, with ROI 1 being the fluorescence of the bleach spot, ROI 2 being the fluorescence of the whole image, and ROI 3 being the Fluorescence of the surrounding unbleached area. The fluorescence was corrected using Equation (2) with *F*_1_ being the fluorescence of ROI 1 and *F*_bg_ being the background fluorescence. The corrected fluorescence curves are fitted according to the modified Soumpasis fit (Equation 3) to determine the time constant *τ* with A and C being the fitting parameters and K_0_ the bleaching parameter. *I*_0_ and *I*_1_ denote the modified Bessel functions of the first kind of order 0 and 1, respectively. The software LAS AF was used for image processing. The parameters for the bleaching process are shown in Table 4.

(2) $$F_{\mathrm{corr}}=\left(F_{1}\left(t\right)-F_{\mathrm{bg}}\right)\;\cdot \frac{F_{2}\left(\mathrm{Pre}-\mathrm{bleach}\right)}{F_{3}\left(t\right)}$$

(3) $$F_{\mathrm{corr}}=A+C\cdot \left(e^{-K_{0}}-1\right)\cdot \left[1-e^{-2\tau /t}\cdot \left(I_{0}\left(2\frac{\tau }{t}\right)+I_{1}\left(2\frac{\tau }{t}\right)\right)\right]$$

**TABLE 4:** Count of images *N* and time between images *t* for each FRAP process.

FITC‐dextran	Parameters	Pre‐bleach	Bleach	Post‐Bleach 1	Post‐Bleach 2	Post‐Bleach 3
4 kDa	*N*(Image)	20	10	15	60	10
	*t*(Image) [min:s:ms]	0:0:147	0:0:147	0:0:147	0:1:0	0:5:0
40 kDa 150 kDa	*N*(Image)	20	10	15	60	50
	*t*(Image) [min:s:ms]	0:0:147	0:0:147	0:0:147	0:1:0	0:5:0

#### 5.6.4 Nanoindentation

The mechanical properties of the microgels were determined using the single indentation method with a Pavone Nanoindenter (Optics11Life). The microgels were fixed on poly‐L‐lysine‐coated wells, which were subsequently filled with Milli‐Q water. A probe with a stiffness of 0.48 N m^−^^1^ and a tip radius of 9 µm was used to indent *n* = 25 microgels with an indentation depth of 1.6 µm. The data were fitted with the Hertz model and analyzed using the Optics11Life Dataviewer software. The microgel cores were measured accordingly after enzyme‐induced shell degradation with a dextranase solution.

#### 5.6.5 Relaxation NMR

T_2_ relaxation NMR measurements were performed with a time domain NMR spectrometer (Bruker minispec mq20) at a frequency of 20 MHz and a temperature of 23°C. For all NMR measurements, the microgels were dispersed in D_2_O. For copper(II)‐induced T_2_ relaxation enhancement, CuSO_4_ (15 g L^−^^1^) was added to the D_2_O.

A CPMG spin‐echo train was measured by using excitation and refocusing RF pulses with a duration of 10.5 µs. A spin echo time of 0.5 ms was employed with 2000 spin echoes and a recycle delay of 3 s. The paramagnetic relaxation enhancement was calculated by determining the difference in T_2_ relaxation times of a sample with and without CuSO_4_.

The T_2_ relaxation times were determined by fitting the CPMG signal decay curve with an exponential decay function (Equation 4).

(4) $$y=y_{0}+A_{1}\cdot e^{-\frac{x}{t_{1}}}+A_{2}\cdot e^{-\frac{x}{t_{2}}}+A_{3}\cdot e^{-\frac{x}{t_{3}}}$$

#### 5.6.6 Rheology

Rheological measurements were performed using a Discovery HR‐3 hybrid rheometer with a flat 20 mm Peltier plate and a gap size of 1 mm. Jammed Microgels (500 µL) were used for the measurements. Shear rate sweep tests were obtained at 1% strain by increasing the shear rate from 0.01 to 100 s^−^^1^. Step strain tests were performed by cycling low strain (1%) and high strain (90%) every 180 s at a constant frequency of 1 Hz. All measurements were carried out at room temperature.

### 5.7 Cell Culture

NHDF were cultured in tissue culture flasks with DMEM supplemented with 10% FCS and 1% ABM at 37°C and 5% CO_2_ up to passage 8. Cellularization of the printed constructs was achieved by seeding 100 µL of cell suspension with varying concentrations on top of the printed constructs. The printed constructs with the cell suspension were incubated for 2 h before 3 mL medium was added.

#### 5.7.1 Live/Dead Staining

The viability of NHDFs incubated with microgels was assessed after 1 and 3 days of incubation time by Live/Dead staining. Microgels (33.3 µL) per well were incubated with cells at a seeding density of 5000 cells cm^−^^2^ in a 24‐well plate. All experimental conditions were performed in triplicate, including microgel‐containing wells and live controls. A dead control was prepared in a single well by exposing cells to 70% ethanol to induce cell death. For Live/Dead staining, a working solution was prepared by adding 20 µL fluorescein diacetate (FDA, 5 mg mL^−^^1^ in acetone) and 20 µL propidium iodide (PI, 0.5 mg mL^−^^1^ in PBS) to 1200 µL phosphate‐buffered saline (PBS). After removal of the culture medium, 200 µL of the staining solution was added to each well and incubated for 5 min at room temperature. PI (red fluorescence) stains the nuclei of membrane‐compromised (dead) cells, whereas FDA (green fluorescence) labels viable cells through intracellular esterase activity. Following incubation, the staining solution was removed and replaced with PBS prior to imaging.

The viability of the cells on top of the constructs was assessed after 2 days of incubation time with a Live/Dead cell imaging kit. The constructs were incubated with live (488/515 nm)/dead (570/602 nm) reagent, diluted in DMEM supplemented with 10% FCS and 1% ABM.

#### 5.7.2 Immunofluorescence Staining

After the cultivation time (2 or 4 days), the culture medium was removed from the samples, and the constructs were washed with PBS for 10 min followed by fixation with PFA for 30 min at RT. After washing twice with PBS, the constructs were incubated with 0.1% Triton X‐100 solution in PBS for 10 min, followed by another washing step with PBS. After blocking the cells with a 4% BSA solution in PBS for 2 h, the cells were stained for F‐actin with Phalloidin‐iFluor 488 (1:1000 in PBS) for 1 h at RT, followed by a washing step with PBS to remove the remaining staining agent. Afterward, DAPI (1:200 in PBS) was added for 20 min to stain the cell nuclei and the microgels. After washing twice with PBS, the constructs were imaged with a confocal microscope.

## Conflicts of Interest

The authors declare no conflicts of interest.

## Supporting information




**Supporting File 1**: adma72780‐sup‐0001‐SuppMat.docx.

**Supporting File 2**: adma72780‐sup‐0002‐VideoS1.mp4.

**Supporting File 3**: adma72780‐sup‐0003‐VideoS2.mp4.

**Supporting File 4**: adma72780‐sup‐0004‐VideoS3.avi.

**Supporting File 5**: adma72780‐sup‐0005‐VideoS4.mov.

**Supporting File 6**: adma72780‐sup‐0006‐VideoS5.mp4.

**Supporting File 7**: adma72780‐sup‐0007‐VideoS6.mp4.

## Data Availability

The data that support the findings of this study are available from the corresponding author upon reasonable request.

## References

[1] F. A. Plamper and W. Richtering , “Functional Microgels and Microgel Systems,” Accounts of Chemical Research 50 (2017): 131–140. 28186408 10.1021/acs.accounts.6b00544

[2] S. Bulut , D. Günther , M. Bund , et al., “Cellular Architects at Work: Cells Building Their Own Microgel Houses,” Advanced Healthcare Materials 13 (2023): 2302957. 10.1002/adhm.202302957 37988182

[3] Y. Kittel , L. P. B. Guerzoni , C. Itzin , et al., “Varying the Stiffness and Diffusivity of Rod‐Shaped Microgels Independently Through Their Molecular Building Blocks,” Angewandte Chemie International Edition 62 (2023): 202309779. 10.1002/anie.202309779 37712344

[4] A. Plucinski , Z. Lyu , and B. V. K. J. Schmidt , “Polysaccharide Nanoparticles: From Fabrication to Applications,” Journal of Materials Chemistry B 9 (2021): 7030–7062. 33928990 10.1039/d1tb00628b

[5] M. Naessens , A. Cerdobbel , W. Soetaert , and E. J. Vandamme , “Leuconostoc Dextransucrase and Dextran: Production, Properties and Applications,” Journal of Chemical Technology & Biotechnology 80 (2005): 845–860.

[6] T. Heinze , T. Liebert , B. Heublein , and S. Hornig , “Functional Polymers Based on Dextran,” Advances in Polymer Science 205 (2006): 199–291.

[7] WHO , The Selection and Use of Essential Medicines 2023. Web Annex A (WHO, 2023) p. 67.

[8] F. Cui , D. Cun , A. Tao , et al., “Preparation and Characterization of Melittin‐Loaded Poly (DL‐Lactic Acid) or Poly (DL‐Lactic‐Co‐Glycolic Acid) Microspheres made by the Double Emulsion Method,” Journal of Controlled Release 107 (2005): 310–319. 16255081 10.1016/j.jconrel.2005.07.001

[9] Y. Ou , S. Cao , Y. Zhang , et al., “Bioprinting Microporous Functional Living Materials from Protein‐Based Core‐Shell Microgels,” Nature Communications 14 (2023): 1–14. 10.1038/s41467-022-35140-5 PMC9852579 36658120

[10] I. Berndt , J. S. Pedersen , and W. Richtering , “Structure of Multiresponsive “Intelligent” Core−Shell Microgels,” Journal of the American Chemical Society 127 (2005): 9372–9373. 15984856 10.1021/ja051825h

[11] C. D. Jones and L. A. Lyon , “Synthesis and Characterization of Multiresponsive Core−Shell Microgels,” Macromolecules 33 (2000): 8301–8306.

[12] A. Pich and W. Richtering , “Microgels by Precipitation Polymerization: Synthesis, Characterization, and Functionalization” Chemical Design of Responsive Microgels (Springer 2010), 1–37.

[13] O. L. J. Virtanen , M. Kather , J. Meyer‐Kirschner , et al., “Direct Monitoring of Microgel Formation During Precipitation Polymerization of N‐Isopropylacrylamide Using In Situ SANS,” ACS Omega 4 (2019): 3690–3699. 31459582 10.1021/acsomega.8b03461 PMC6648459

[14] X. Wu , R. H. Pelton , A. E. Hamielec , D. R. Woods , and W. McPhee , “The Kinetics of Poly(N‐isopropylacrylamide) Microgel Latex Formation,” Colloid & Polymer Science 272 (1994): 467–477.

[15] I. Varga , T. Gilányi , R. Mészáros , G. Filipcsei , and M. Zrínyi , “Effect of Cross‐Link Density on the Internal Structure of Poly(N‐isopropylacrylamide) Microgels,” The Journal of Physical Chemistry B 105 (2001): 9071–9076.

[16] Y. Nishizawa , K. Honda , M. Karg , and D. Suzuki , “Controlling the Shell Structure of Hard Core/Hydrogel Shell Microspheres,” Colloid and Polymer Science 300 (2022): 333–340.

[17] J. T. Wang , J. Wang , and J. J. Han , “Fabrication of Advanced Particles and Particle‐Based Materials Assisted by Droplet‐Based Microfluidics,” Small 7 (2011): 1728–1754. 21618428 10.1002/smll.201001913

[18] L. Kong , A. Levin , Z. Toprakcioglu , et al., “Lipid‐Stabilized Double Emulsions Generated in Planar Microfluidic Devices,” Langmuir 36 (2020): 2349–2356. 32045250 10.1021/acs.langmuir.9b03622

[19] S. Guo , G. Kang , D. T. Phan , M. N. Hsu , Y. C. Por , and C. H. Chen , “Polymerization‐Induced Phase Separation Formation of Structured Hydrogel Particles via Microfluidics for Scar Therapeutics,” Scientific Reports 8 (2018): 1–10. 29396452 10.1038/s41598-018-20516-9 PMC5797090

[20] B. U. Moon , N. Abbasi , S. G. Jones , D. K. Hwang , and S. S. H. Tsai , “Water‐in‐Water Droplets by Passive Microfluidic Flow Focusing,” Analytical Chemistry 88 (2016): 3982–3989. 26959358 10.1021/acs.analchem.6b00225

[21] I. Ziemecka , V. Van Steijn , G. J. M. Koper , M. T. Kreutzer , and J. H. Van Esch , “All‐Aqueous Core‐Shell Droplets Produced in a Microfluidic Device,” Soft Matter 7 (2011): 9878–9880.

[22] T. Y. Lee , C. A. Guymon , E. Jönsson , and C. Hoyle , “The Effect of Monomer Structure on Oxygen Inhibition of (Meth)Acrylates Photopolymerization” Polymer 45 (2004), 6155–6162.

[23] S. C. Ligon , B. Husár , H. Wutzel , R. Holman , and R. Liska , “Strategies to Reduce Oxygen Inhibition in Photoinduced Polymerization,” Chemical Reviews 114 (2014): 557–589. 24083614 10.1021/cr3005197

[24] L. Gou , B. Opheim , C. N. Coretsopoulos , and A. B. Scranton , “Consumption of the Molecular Oxygen in Polymerization Systems Using Photosensitized Oxidation of Dimethylanthracene,” Chemical Engineering Communications 193 (2006): 620–627.

[25] D. Dendukuri , S. S. Gu , D. C. Pregibon , T. A. Hatton , and P. S. Doyle , “Stop‐Flow Lithography in a Microfluidic Device,” Lab on a Chip 7 (2007): 818. 17593999 10.1039/b703457a

[26] J. R. Tumbleston , D. Shirvanyants , N. Ermoshkin , et al., “Continuous Liquid Interface Production of 3D Objects,” Science 347 (2015): 1349–1352. 25780246 10.1126/science.aaa2397

[27] D. Debroy , J. Oakey , and D. Li , “Interfacially‐Mediated Oxygen Inhibition for Precise and Continuous Poly(ethylene glycol) Diacrylate (PEGDA) Particle Fabrication,” Journal of Colloid and Interface Science 510 (2018): 334–344. 28961432 10.1016/j.jcis.2017.09.081

[28] C. Decker , “Kinetic Study and New Applications of UV Radiation Curing,” Macromolecular Rapid Communications 23 (2002): 1067–1093.

[29] J.‐P. Fouassier , Photoinitiation, Photopolymerization, and Photocuring: Fundamentals and Applications (Hanser, 1995) 375.

[30] K. Krutkramelis , B. Xia , and J. Oakey , “Monodisperse Polyethylene Glycol Diacrylate Hydrogel Microsphere Formation by Oxygen‐Controlled Photopolymerization in a Microfluidic Device,” Lab on a Chip 16 (2016): 1457–1465. 26987384 10.1039/c6lc00254d PMC4829474

[31] S. Bulut , S. Jung , T. Bissing , et al., “Tuning the Porosity of Dextran Microgels with Supramacromolecular Nanogels as Soft Sacrificial Templates,” Small 19 (2023): 2303783. 10.1002/smll.202303783 37434076

[32] J. Ambati , C. S. Canakis , J. W. Miller , et al., “Diffusion of High Molecular Weight Compounds through Sclera,” Investigative Ophthalmology & Visual Science 41 (2000): 1181–1185. 10752958

[33] D. M. Soumpasis , “Theoretical Analysis of Fluorescence Photobleaching Recovery Experiments,” Biophysical Journal 41 (1983): 95–97. 6824758 10.1016/S0006-3495(83)84410-5 PMC1329018

[34] D. E. Demco and A. Pich , “Structure and Dynamics of Temperature‐Responsive Microgels and Hydrogels by NMR Spectroscopy, Relaxometry, and Diffusometry,” Macromolecular Chemistry and Physics 224 (2023): 2200410.

[35] S.‐H. Jung , S. Bulut , L. P. B. Busca Guerzoni , et al., “Fabrication of pH‐Degradable Supramacromolecular Microgels with Tunable Size and Shape via Droplet‐Based Microfluidics,” Journal of Colloid and Interface Science 617 (2022): 409–421. 35279576 10.1016/j.jcis.2022.02.065

[36] W. N. E. van Dijk‐Wolthuis , J. J. K. den Bosch , A. A. van der Kerk‐van Hoof , and W. E. Hennink , “Reaction of Dextran with Glycidyl Methacrylate: An Unexpected Transesterification,” Macromolecules 30 (1997): 3411–3413.

