# Supplementary material for: Oxygen‐Inhibition Driven Compartmentalization of Dextran Microgels: Toward Fusible Colloidal Biomaterial Inks for 3D Printing
Source: Adv Mater. 2026 Mar 14;38(20):e19972. doi: 10.1002/adma.202519972 (PMC13054202; doi:10.1002/adma.202519972)
Supplement: Supplementary file 1 — Supporting File 1: adma72780‐sup‐0001‐SuppMat.docx. [file ADMA-38-e19972-s005.docx]

Supporting Information

**Oxygen-inhibited compartmentalization of dextran-based microgels via droplet-based microfluidics for 3D printing applications**

Selin Bulut, Thomas Bissing, Tudor Lile, Hannah Küttner, Daniel Günther, Cédric Bergerbit, Dan E. Demco, Laura De Laporte, and Andrij Pich*


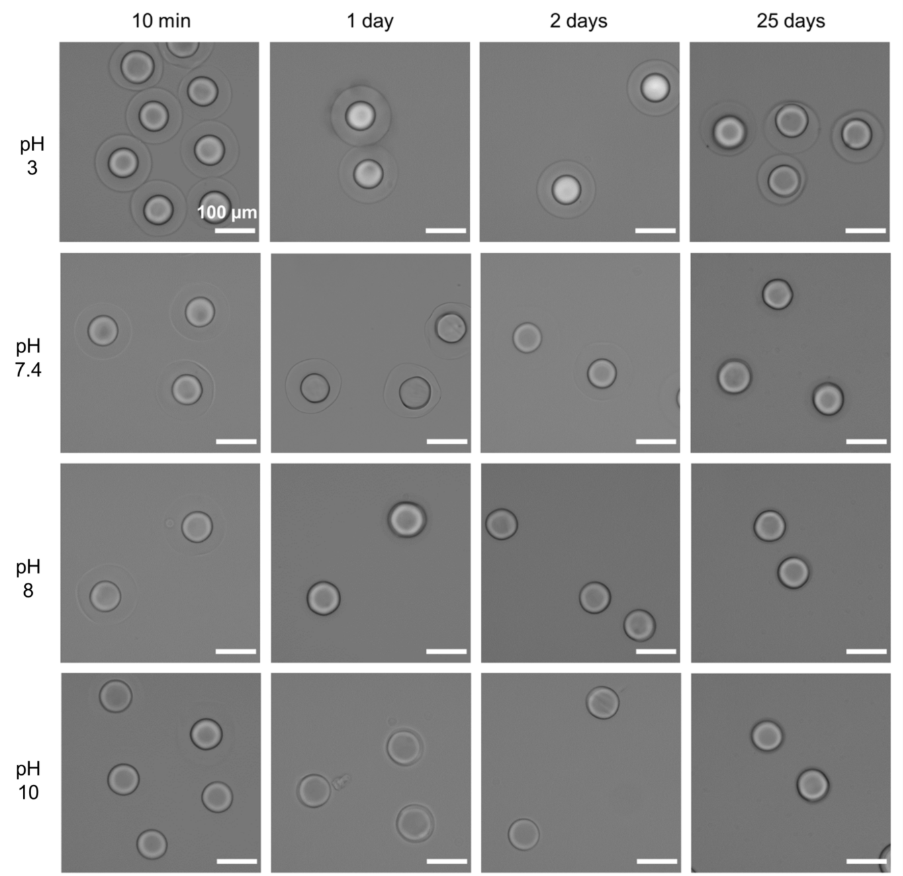
**Figure S1.** Degradation kinetics of Core-Shell Microgels at different pH values over several days. Scale bars represent 100 µm.


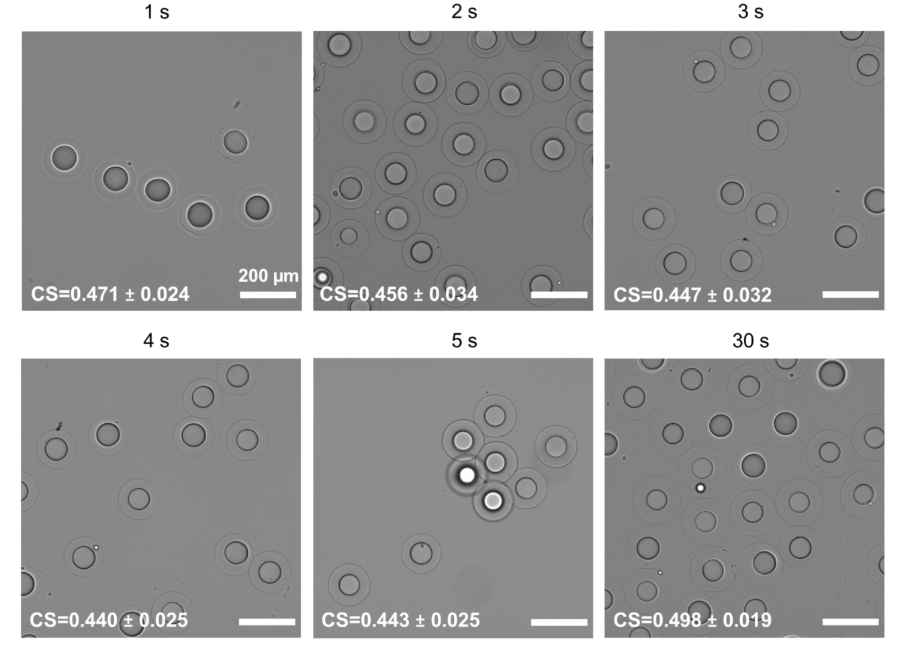


**Figure S2.** Core–shell formation of batch microgels at short UV irradiation times (1–30 s) produced with 1 g L⁻¹ LAP.


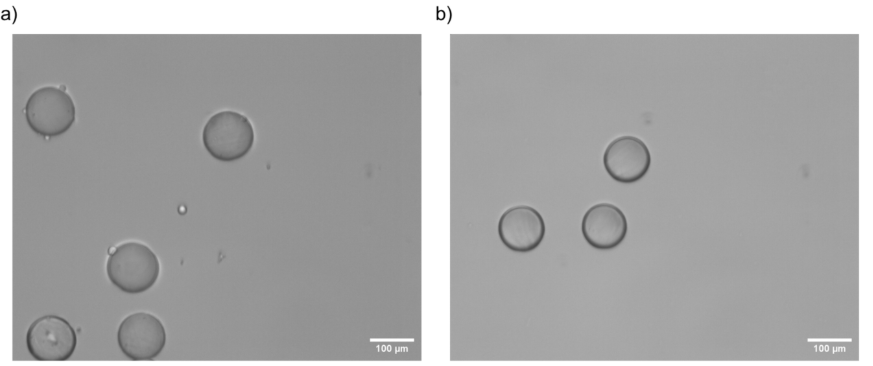


**Figure S3.** Brightfield Microscopy images of a) dex-MA microgels (10 g L⁻¹ LAP, 30 s UV irradiation). b) dex-MA microgels (10 g L⁻¹ LAP, 1 h UV irradiation) produced with the batch method. High CS values of 0.12 ± 0.01 (exact values: 0.121 ± 0.019 and 0.116 ± 0.007, respectively) were observed for 30 s or 1 h UV exposure.


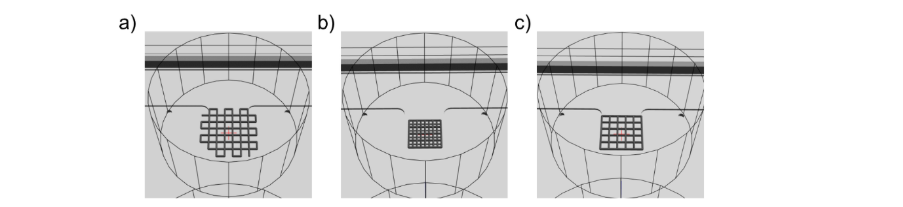


**Figure S4.** CAD drawings of the printed constructs. a) open-edged grid-geometry with 4 mm² gaps b) close-edge grid-geometry with alternating layers with 1 mm² and 4 mm² gaps (c) close-edged grid-geometry with 4 mm² gaps.


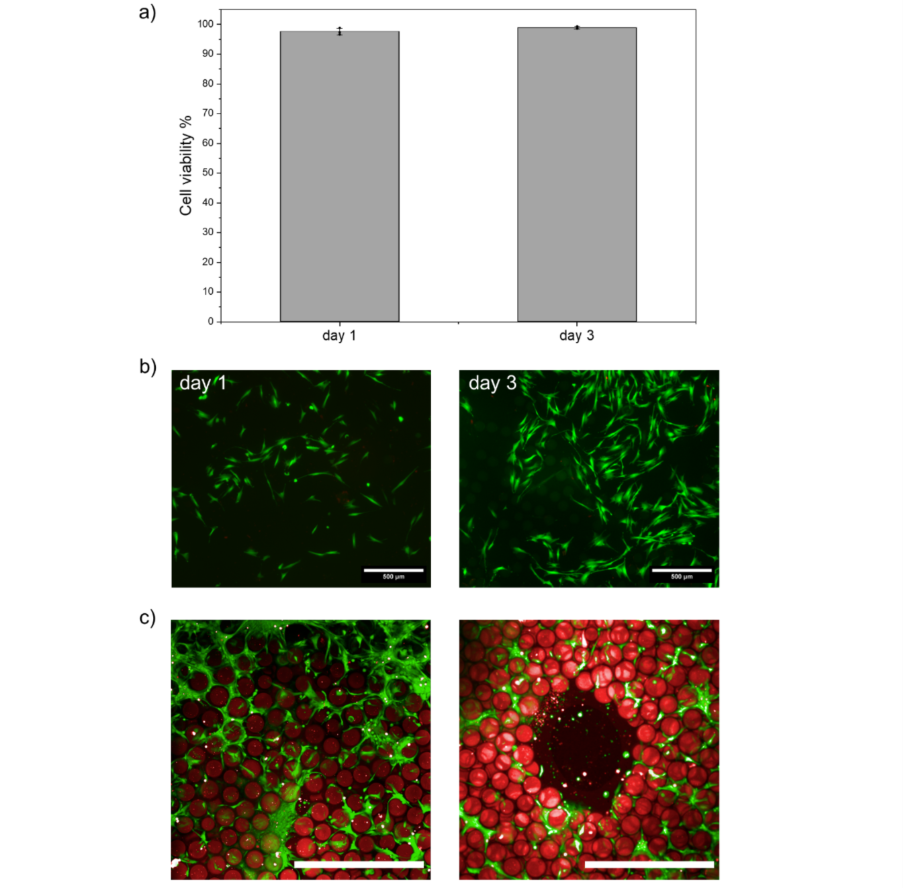


**Figure S5**. a) Live/Dead assessment of NHDF viability mixed with core-shell microgels. Quantification of cell viability after 1 and 3 days of culture in %. Data presented as mean ± SD (n = 3 independent replicates). b) Fluorescence images showing live cells (green) and dead cells (red) at days 1 and 3. Scale bars are 500 µm. c) CLSM images of a Live/Dead stained cellularized construct after 2 days of incubation. A region without a void (left image), and a region with a void (right image). Live cells are shown in green, while the red signal indicates dead cells and stained microgels. Scale bars are 500 µm.


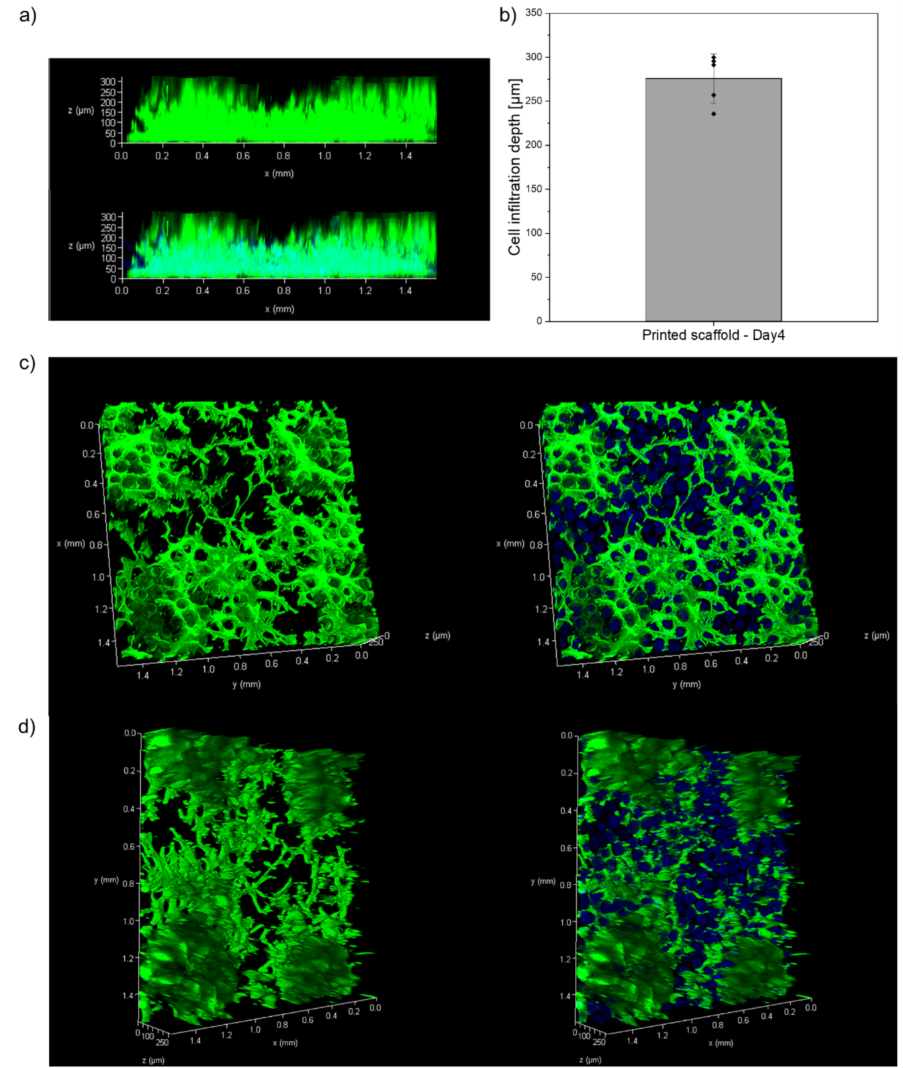
**Figure S6.** NHDF presence throughout the construct, starting at the bottom of the 3D-printed core-shell microgel scaffolds after 4 days of incubation. a) Orthogonal XZ projections of phalloidin-stained NHDFs (green), cell nuclei and microgels (blue), and merged channels, revealing cell penetration in the interior of the construct. b) Quantification of maximum cell infiltration depth determined from orthogonal projections (n = 5 independent measurement positions). Representative 3D volume rendering c) (bottom view) and d) (top view) illustrating cellular distribution within the interconnected pore architecture of the printed scaffold.


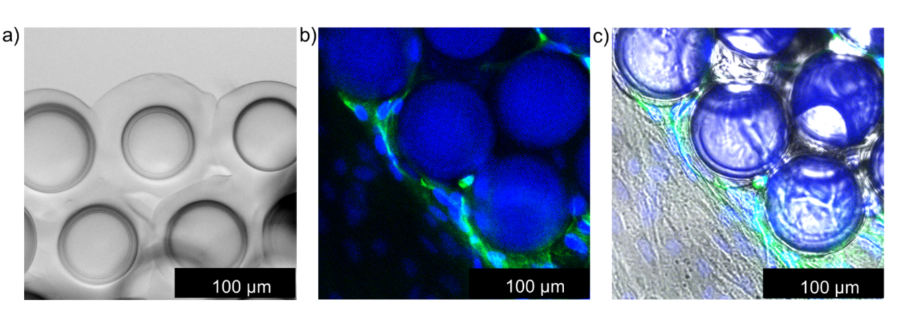


**Figure S7.** a) Brightfield image of annealed core-shell microgels after printing in PBS. b) Confocal image of cellularized printed constructs after 4 days of incubation. The actin cytoskeleton of NHDFs is stained with Phalloidin (green), while nuclei and microgels are stained with DAPI (blue) c) Overlay of fluorescence and brightfield images after 4 days of culture, illustrating cell attachment along the microgel interfaces and within the interstitial spaces of the construct. Scale bars are 100 µm.
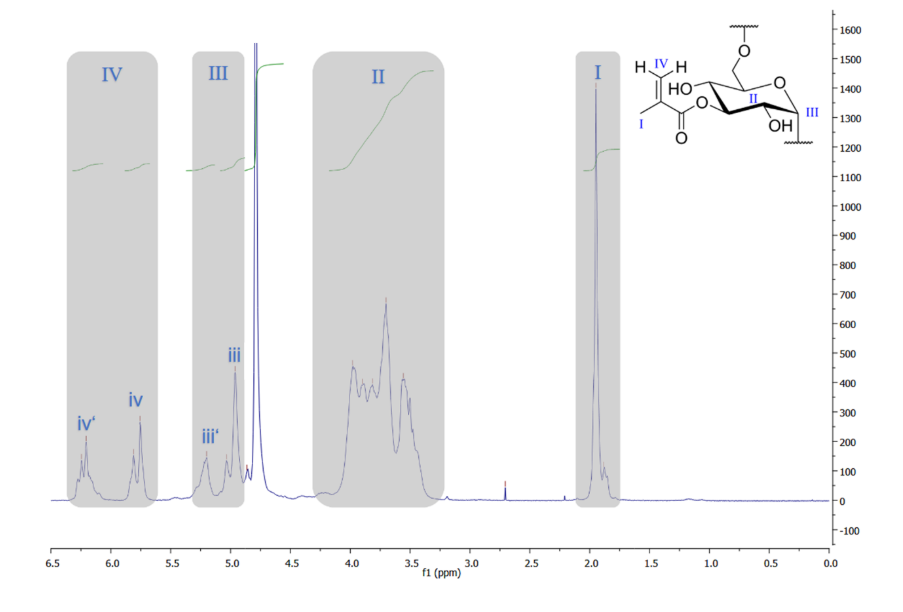


**Figure S8.** ^1^H NMR spectrum of dextran-methacrylate.

**Supporting Video 1.** Degradation of core-shell microgels loaded with FITC-dextran upon pH change and release of the payload.

**Supporting Video 2.** Enzymatic degradation of core-shell microgels loaded with FITC-dextran upon addition of a dextranase solution (2 mg mL⁻¹, 10 U mg⁻¹) and release of the payload.

**Supporting Video 3.** Reswelling of the printed construct in water.

**Supporting Video 4.** Stability test of the 3D printed construct in water.

**Supporting Video 5.** Three-dimensional projection of the confocal z-stack of the printed microgel construct visualizing the construct boundary (bottom to top view).

**Supporting Video 6.** Three-dimensional projection of the confocal z-stack of the printed microgel construct (top to bottom view).
